# CD40 signal rewires fatty acid and glutamine metabolism for stimulating macrophage anti-tumorigenic functions

**DOI:** 10.1038/s41590-023-01430-3

**Published:** 2023-02-23

**Authors:** Pu-Ste Liu, Yi-Ting Chen, Xiaoyun Li, Pei-Chun Hsueh, Sheue-Fen Tzeng, Hsi Chen, Pei-Zhu Shi, Xin Xie, Sweta Parik, Mélanie Planque, Sarah-Maria Fendt, Ping-Chih Ho

**Affiliations:** 1 Institute of Cellular and System Medicine, National Health Research Institute, Miaoli, Taiwan; 2 Department of Fundamental Oncology, Faculty of Biology and Medicine, University of Lausanne, Lausanne, Switzerland; 3 Ludwig Lausanne Branch, Lausanne, Switzerland; 4 School of Life Science, Shaoxing University, Shaoxing, People’s Republic of China; 5 Laboratory of Cellular Metabolism and Metabolic Regulation, Center for Cancer Biology, VIB, Leuven, Belgium; 6 Laboratory of Cellular Metabolism and Metabolic Regulation, Department of Oncology, KU Leuven and Leuven Cancer Institute (LKI), Leuven, Belgium

**Keywords:** Biochemistry, Tumour immunology, Monocytes and macrophages

## Abstract

Exposure of lipopolysaccharide triggers macrophage pro-inflammatory polarization accompanied by metabolic reprogramming, characterized by elevated aerobic glycolysis and a broken tricarboxylic acid cycle. However, in contrast to lipopolysaccharide, CD40 signal is able to drive pro-inflammatory and anti-tumorigenic polarization by some yet undefined metabolic programming. Here we show that CD40 activation triggers fatty acid oxidation (FAO) and glutamine metabolism to promote ATP citrate lyase-dependent epigenetic reprogramming of pro-inflammatory genes and anti-tumorigenic phenotypes in macrophages. Mechanistically, glutamine usage reinforces FAO-induced pro-inflammatory and anti-tumorigenic activation by fine-tuning the NAD^+^/NADH ratio via glutamine-to-lactate conversion. Genetic ablation of important metabolic enzymes involved in CD40-mediated metabolic reprogramming abolishes agonistic anti-CD40-induced antitumor responses and reeducation of tumor-associated macrophages. Together these data show that metabolic reprogramming, which includes FAO and glutamine metabolism, controls the activation of pro-inflammatory and anti-tumorigenic polarization, and highlight a therapeutic potential of metabolic preconditioning of tumor-associated macrophages before agonistic anti-CD40 treatments.

## Abstract (web summary)

Unlike metabolic reprogramming that is characteristic of macrophage inflammatory polarization responses to lipopolysaccharide and TLR4 stimulation, the metabolism underlying inflammatory responses to CD40 signaling is not well characterized. Here the authors show CD40 signaling drives fatty acid oxidation and glutamine metabolism resulting in regulation of the NAD^+^/NADH ratio, which in turn promotes antitumor and pro-inflammatory macrophage functions.

## Main

Tumor-associated macrophages (TAMs), one of the most abundant immune subsets in the tumor microenvironment (TME), have been shown to modulate the formation of an immunosuppressive TME and impair host antitumor responses through pro-tumorigenic activities^1,2^. Reeducation of TAMs by targeting cytokine signaling and potentially other tumor microenvironmental factors to confer anti-tumorigenic features is considered an attractive strategy to promote host antitumor immunity. In tumors, the education of TAMs to promote pro-tumorigenic polarization is dependent on the cytokine milieu; however, the metabolic context of the TME has also been reported to orchestrate functional polarization of TAMs^2,3^. Similarly to alternatively activated (M2) macrophages, which are induced by interleukin (IL-)4 and IL-13, pro-tumorigenic TAMs increase fatty acid uptake and glutamine metabolism and display high oxidative phosphorylation (OXPHOS) rates that drive pro-tumorigenic phenotypes, thus supporting tumor growth^4–8^. By contrast, anti-tumorigenic TAMs undergo metabolic changes similar to those that occur in classically activated (M1) macrophages, including an increment in aerobic glycolysis, a reduction of oxidative metabolism and a disruption of the tricarboxylic acid (TCA) cycle^9–12^. Even though we have an understanding of the metabolic reprogramming underlying macrophage polarization in response to conventional stimuli, such as lipopolysaccharide (LPS) and IL-4/IL-13, it is unclear whether metabolic machinery contributes to TAM reeducation. Also, it is unclear whether macrophages can undergo pro-inflammatory/anti-tumorigenic polarization in response to metabolic stress, such as glucose depletion, lipid overload and hypoxic conditions.

CD40 is strongly expressed by macrophages as well as other antigen-presenting cells (APCs), and functions as a receptor for the CD40 ligand (CD40L), which is abundant on activated CD4^+^ T cells. The CD40–CD40L interaction triggers several molecular events, including the activation of nuclear factor-kappa B, phosphoinositide 3-kinase and mitogen-activated protein kinase, that modulate the cellular behavior of APCs in a cell-type-dependent manner^13^. In macrophages, the activation of CD40-mediated signaling by agonistic anti-CD40 monoclonal antibodies stimulates the expression of pro-inflammatory marker genes. Moreover, treating tumor-bearing mice with agonistic anti-CD40 monoclonal antibodies, including clone FGK45, drives reeducation of TAMs toward an anti-tumorigenic phenotype and induces macrophage-dependent antitumor responses^14–17^. However, the metabolic programs involved in CD40-mediated activation of pro-inflammatory and anti-tumorigenic macrophage polarization are unclear. Moreover, while LPS inactivates AMP-activated protein kinase (AMPK)^18^, CD40 signaling has been reported to stimulate the phosphorylation of AMPK at Thr172 (a critical activation residue) and to support survival of macrophages under serum-deprived conditions^6,19^. These differences in AMPK regulation suggest that CD40 signaling might use different metabolic programs compared to those induced by LPS.

Here, we report that CD40 signaling promotes FAO and glutamine metabolism to trigger the epigenetic reprogramming required for pro-inflammatory/anti-tumorigenic polarization. Mechanistically, CD40 activation increases histone acetylation at promoters and enhancers of pro-inflammatory marker genes by using acetyl-CoA generated by FAO. CD40 signaling also triggers glutamine-to-lactate conversion. Intriguingly, the production of lactate from glutamine sustains FAO by fine-tuning the NAD^+^/NADH ratio. This study reveals that FAO and glutamine metabolism, normally associated with M2 polarization, can be exploited for orchestrating pro-inflammatory and anti-tumorigenic macrophage polarization and CD40-triggered pro-inflammatory activation does not interrupt the TCA cycle. Moreover, our findings highlight that the preconditioning of the TME metabolic milieu is a critical prerequisite for agonistic anti-CD40 monoclonal antibodies on eliciting antitumor responses.

## Results

### OXPHOS supports CD40-mediated anti-tumorigenic polarization

**Fig. 1:**
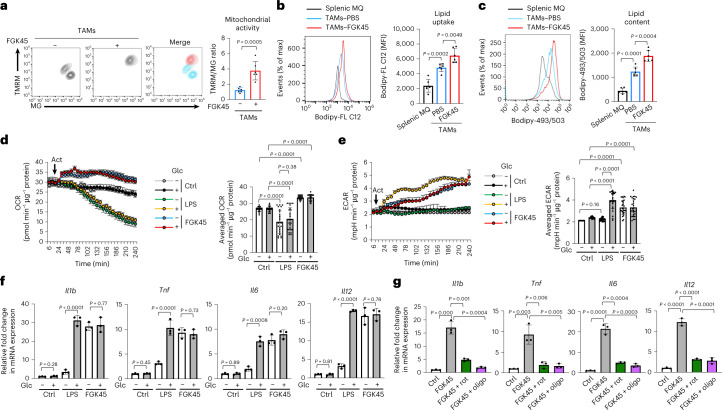
CD40 signal stimulates unconventional metabolic reprogramming. **a**, Mitochondrial mass and membrane potential in TAMs isolated from tumor-bearing mice treated with or without FGK45 monoclonal antibody (mAb) were determined by MG and TMRM, respectively. The ratio of TMRM to MG in TAMs was quantified. **b**,**c**, Representative histogram and quantitative analysis of the mean fluorescence intensity (MFI) of Bodipy-FL C12 (**b**) and Bodipy-493/503 (**c**) in splenic macrophages (MQ), TAMs isolated from tumor-bearing mice treated with PBS or FGK45 mAb. **d**, Real-time changes of the OCR and the average of basal OCR of BMDMs stimulated with control vehicle (Ctrl), LPS or FGK45 mAb under glucose-replete or glucose-deplete conditions. **e**, Real-time changes of the ECAR and the average of basal ECAR of BMDMs stimulated with Ctrl, LPS or FGK45 mAb under glucose-replete or glucose-deplete conditions. Arrowheads indicate the time of LPS or FGK45 mAb treatment. **f**, Relative fold changes in mRNA expression of pro-inflammatory marker genes in BMDMs stimulated with Ctrl, LPS or FGK45 mAb under indicated conditions. **g**, qPCR analysis of mRNA expression of pro-inflammatory marker genes in BMDMs stimulated with FGK45 mAb in the absence or presence of rotenone (rot) or oligomycin (oligo) under glucose-deplete media. **a**–**c**, Representative data from two individual experiments. Each point represents biologically independent samples (*n* = 6 mice per group). Data in bar graphs are presented as the mean ± s.d. **d**–**g**, Representative data from three individual experiments with *n* = 3 per group. Data are the mean ± s.d. All data were analyzed by a two-tailed Student’s *t*-test. Source data

**Extended Data Fig. 1:**
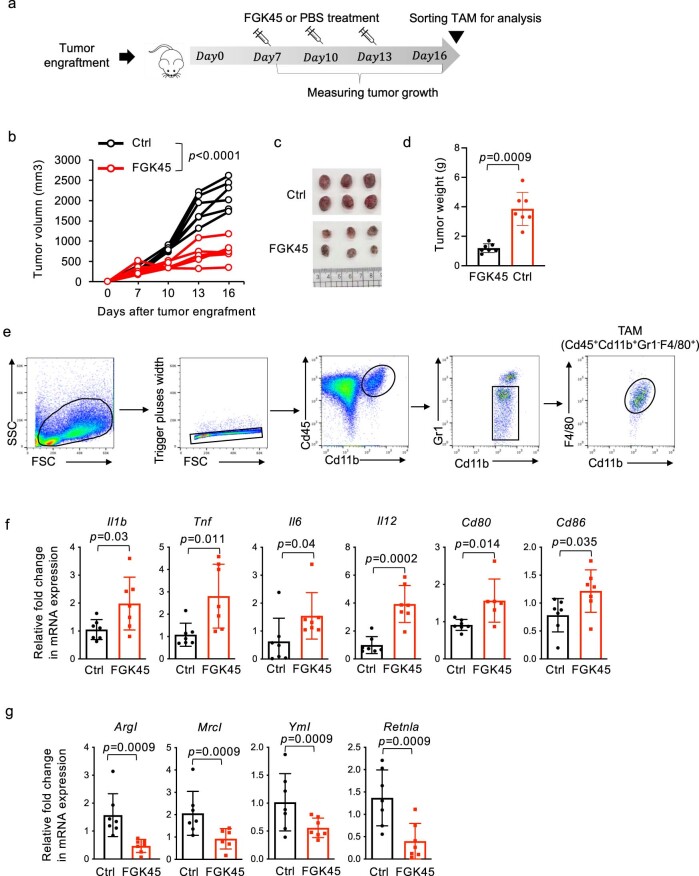
CD40 mAb induces anti-tumor responses. (**a**) Illustration of the design for FGK45 treatment in tumor-bearing mice. (**b–d**) Tumor growth (**b**), representative appearances (**c**), and tumor weight (**d**) of tumors from indicated treatment groups (n = 6 mice per group). Data are representative of 2 independent experiments. (**e**) Representative gating strategy of flow cytometry to isolate tumor-associated macrophages (TAMs). (**f, g**) qPCR analysis of mRNA expression of proinflammatory marker genes (**f**) and anti-inflammatory genes (**g**) in TAMs isolated from tumor-bearing mice treated with control vehicle (Ctrl) or FGK45 mAb. (**f, g**) Biologically independent samples were pooled from two independent experiments (n = 7 mice per group). All data are presented as mean ± SD and analyzed by a two-tailed Student’s t-test. Source data

**Extended Data Fig. 2:**
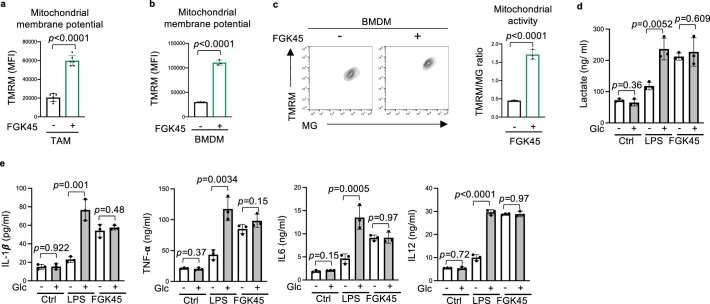
CD40 signal reprogram macrophage OXPHOS and mitochondrial activity. (**a**) Quantitative result of mitochondrial membrane potential in TAMs isolated from tumor-bearing mice treated with or without FGK45 mAb, which was determined by tetramethylrhodamine (TMRM). Dots represent individual mouse. (**b**) Quantitative result of mitochondrial membrane potential in BMDMs stimulated with or without FGK45 mAb, which was determined by tetramethylrhodamine (TMRM). (**c**) Mitochondrial mass and membrane potential in BMDMs stimulated with or without FGK45 mAb were determined by MitoTracker Green (MG) and tetramethylrhodamine (TMRM), respectively. The representative flow plots (left) and the quantitative result of MDR to MG ratio (right) in. (**d–e**) Extracellular lactate levels (**d**) and the production of proinflammatory cytokines (**e**) in BMDMs stimulated with or without FGK45 mAb or LPS under glucose-replete or -deplete conditions were determined by ELISA. (**a**) Data are representative of two independent experiments. Dots represent individual mouse (n = 6 mice per group). (**b–e**) Data are representative of three independent experiments (n = 3 per group). All data are presented as mean ± SD and analyzed by a two-tailed Student’s t-test. Source data

To analyze TAM activation and cell metabolic state in response to CD40 stimulation, we engrafted the YUMM1.7 melanoma cell line^20^ into wild-type mice followed by either control vehicle (phosphate-buffered saline, PBS) or agonistic anti-CD40 monoclonal antibody (FGK45) treatments (Extended Data Fig. 1a). As previously reported^14,15^, agonistic anti-CD40 monoclonal antibodies effectively suppressed melanoma growth, which was accompanied with a strong induction of expression of anti-tumorigenic genes, but reduced levels of genes related to pro-tumorigenic and tissue-repairing processes in TAMs (Extended Data Fig. 1b–g). Moreover, FGK45 affected mitochondrial mass and membrane potential in TAMs as measured through MitoTracker Green (MG) and tetramethylrhodamine (TMRM) staining, respectively. TAMs from mice treated with FGK45 increased TMRM staining (Extended Data Fig. 2a) and showed a higher TMRM/MG ratio (Fig. 1a), an indicator of mitochondrial activity per mitochondrial mass. Similarly, FGK45 treatment increased TMRM staining and TMRM/MG ratio in bone marrow-derived macrophages (BMDMs; Extended Data Fig. 2b,c), indicating that CD40 signaling stimulates mitochondrial activity in macrophages. We further found that FGK45 increased lipid uptake and lipid content (Fig. 1b,c). An increase in mitochondrial activity and lipid uptake is considered a metabolic signature of anti-inflammatory and pro-tumorigenic activation of macrophages^4,7^; however, FGK45 treatment stimulated an anti-tumorigenic activity in TAMs. These results led us to speculate that CD40 signaling may trigger pro-inflammatory and anti-tumorigenic macrophage polarization via an unexplored metabolic remodeling program controlled by mitochondrial metabolism. To verify this, we first determined oxygen consumption rate (OCR) and extracellular acidification rate (ECAR) in BMDMs treated with LPS or FGK45 in the presence or absence of glucose by using the Seahorse extracellular flux analyzer. LPS treatment reduced OCR as reported previously^4^. However, in line with the increase in mitochondrial activity, FGK45 treatment augmented OCR in BMDMs (Fig. 1d). Of note, in contrast to LPS treatment, which did not increase ECAR and lactate production in glucose-deprived conditions, FGK45 stimulation increased ECAR and lactate production in BMDMs in a glucose-independent manner (Fig. 1e and Extended Data Fig. 2d). Moreover, unlike LPS, FGK45 promoted the expression of pro-inflammatory marker genes and the production pro-inflammatory cytokines in glucose-deprived conditions (Fig. 1f and Extended Data Fig. 2e). Intriguingly, inhibition of OXPHOS with rotenone or oligomycin impaired FGK45-mediated induction of pro-inflammatory marker genes (Fig. 1g), suggesting that mitochondrial metabolism, rather than glycolysis, supports pro-inflammatory macrophage activation mediated by CD40 signaling pathway. Altogether, these results demonstrate that CD40 signaling can reprogram a previously unreported, OXPHOS-dependent metabolic pathway to support pro-inflammatory and anti-tumorigenic macrophage polarization.

### CD40 signaling boosts fatty acid oxidation

**Fig. 2:**
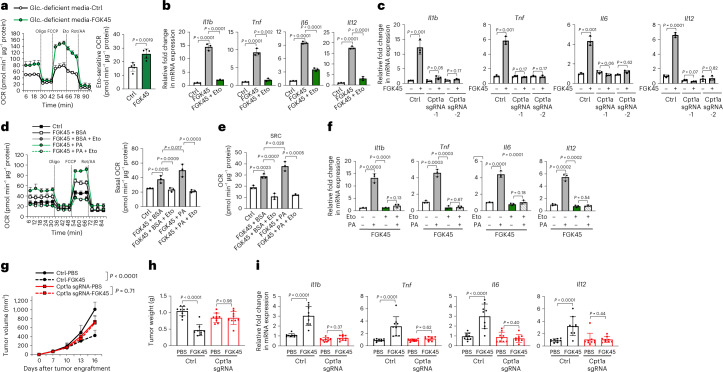
Fatty acid oxidation supports CD40-driven pro-inflammatory and anti-tumorigenic polarization. **a**, The real-time changes of OCR and the average of etomoxir (Eto)-sensitive OCR of BMDMs stimulated with either control vehicle (Ctrl) or FGK45 mAb under glucose-deplete conditions in the basal state and following the additions of oligomycin (Oligo), FCCP, Eto and rotenone + antimycin A (Rot/AA). **b**, qPCR analysis of mRNA expression of pro-inflammatory marker genes in BMDMs stimulated with either Ctrl or FGK45 mAb in the absence or presence of Eto under glucose-deplete media. **c**, qPCR analysis of mRNA expression of mRNA expression of pro-inflammatory marker genes in Cas9-expressing BMDMs transduced with lentivirus expressing either control gRNA (Ctrl) or gRNAs targeting CPT1a (Cpt1a sgRNA-1 and sgRNA-2) stimulated with or without FGK45 mAb in glucose-deplete media. **d**,**e**, The real-time changes of OCR (**d**, left), the average of basal OCR (**d**, right) and SRC (**e**) of BMDMs loaded with BSA or palmitic acid (PA)-conjugated BSA and then followed by stimulation with Ctrl or FGK45 mAb in the presence or absence of etomoxir in glucose-deplete media. **f**, qPCR analysis of mRNA expression of pro-inflammatory genes in BMDMs loaded with BSA or PA-conjugated BSA and then followed by stimulation with Ctr) or FGK45 mAb in the presence or absence of etomoxir in glucose-deplete media. **g**,**h**, Tumor growth (**g**) and tumor weight (**h**) in tumor-bearing mice transplanted with either indicated bone marrows and then treated with either PBS or FGK45 mAb. **i**, Relative fold change in mRNA expression of pro-inflammatory marker genes in TAMs isolated from tumor-bearing mice transplanted with either indicated bone marrows and then treated with either PBS or FGK45 mAb. **a**–**f**, Data are representative of three individual experiments with *n* = 5 per group in **a** and *n* = 3 per group in **b**–**f**. **g**–**i**, Data represent pooled biologically independent samples from two experiments (*n* = 9 mice per group). Data are the mean ± s.d. Statistical analysis was determined by two-tailed Student’s *t*-test. Source data

**Extended Data Fig. 3:**
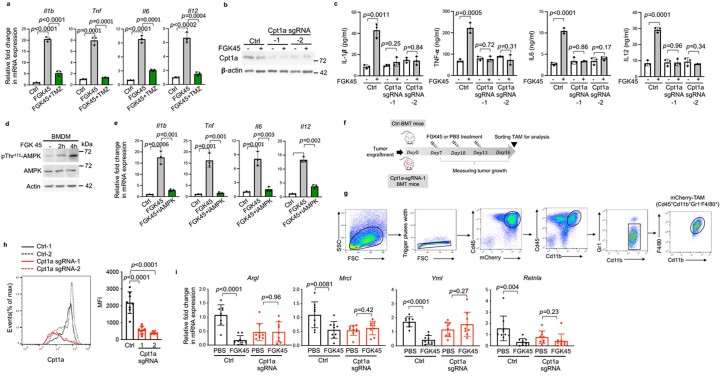
FAO supports CD40-driven macrophage polarization. (**a**) qPCR analysis of mRNA expression of proinflammatory marker genes in BMDMs stimulated with or without FGK45 mAb in the absence or presence of trimetazidine (TMZ) under glucose-deplete media. (**b, c**) Representative immunoblots of CPT1a and β-actin (**b**) and the production of proinflammatory cytokines (**c**) in Cas9-expressing BMDMs harboring control sgRNA (Ctrl) and CPT1A sgRNAs (Cpt1a sgRNA-1 and -2) stimulated with or without FGK45 mAb. (**d**) Representative immunoblots of indicated proteins in BMDMs stimulated with or without FGK45 mAb. (**e**) qPCR analysis of mRNA expression of proinflammatory marker genes in BMDMs stimulated with or without FGK45 in the absence or presence of AMPK inhibitor (iAMPK; Dorsomorphin, 20 μM) under glucose-deplete media. (**f**) Illustration of the design for bone marrow transplantation in mice follwed with tumor engraftment and then FGK45 treatment. (**g**) Representative gating strategy of flow cytometry to isolate tumor-associated macrophages (TAMs). (**h**) Representative flow plot (left) and quantitative results (right) of CPT1a expression in TAMs isolated from tumor-bearing mice transplantate with indicated bone marrows. (**i**) qPCR analysis of mRNA expression of anti-inflammatory marker genes in TAMs isolated from indicated tumor-bearing treated with control vehicle (PBS) or FGK45 mAb. (**a–e**) Representative data from three individual experiments (n = 3 per group). (**h–i**) Representative data from two individual experiments. Dots represent individual mouse (**h**, n = 8; **i**, n = 9 mice per group). All data are presented as mean ± SD and analyzed by a two-tailed Student’s t-test. Source data

Because FGK45 monoclonal antibodies increase lipid uptake and OXPHOS activity in macrophages, we wondered whether CD40 signaling could enhance OXPHOS via FAO. Then, we treated FGK45-stimulated BMDMs with etomoxir, an inhibitor of FAO^4,21^ and observed that FGK45 treatment increased FAO-dependent OCR in BMDMs and etomoxir abolished FGK45-mediated induction of pro-inflammatory marker genes (Fig. 2a,b). Since etomoxir is known to have an off-target effect on mitochondrial electron transport chain activity and OXPHOS^22,23^, we next tested trimetazidine, an FAO inhibitor targeting 3-ketoacyl CoA thiolase, and confirmed that trimetazidine also hampered FGK45-mediated induction of pro-inflammatory marker genes (Extended Data Fig. 3a). To further validate the contribution of FAO, we transduced BMDMs generated from *LysM*-Cre Cas9 mice^24^ with lentiviruses expressing a single-guide RNA (sgRNA) targeting the *Cpt1a* gene encoding carnitine palmitoyltransferase 1a, a rate-limiting enzyme of FAO. The expression of *Cpt1a*-targeting gRNAs effectively reduced Cpt1a protein expression (Extended Data Fig. 3b). In Cpt1a-deficient BMDMs, FGK45 failed to induce expression of pro-inflammatory marker genes and cytokines (Fig. 2c and Extended Data Fig. 3c), indicating that FAO supports CD40-triggered pro-inflammatory activation. Next, we found that BMDMs pre-loaded with bovine serum albumin (BSA)–palmitate conjugates showed a robust increase in FGK45-induced OCR and spare respiratory capacity (SRC), in an etomoxir-dependent manner (Fig. 2d,e). In addition, palmitate pre-loaded macrophages expressed high levels of pro-inflammatory marker genes in response to FGK45, but this transcriptional enhancement was abolished by etomoxir (Fig. 2f).

Unlike LPS, which inactivates AMPK^18^, CD40 signaling promoted AMPK activation in BMDMs (Extended Data Fig. 3d)^6^. Moreover, dorsomorphin, an AMPK inhibitor, suppressed FGK45-mediated induction of pro-inflammatory marker genes (Extended Data Fig. 3e), indicating that CD40 signaling may support pro-inflammatory polarization via AMPK-dependent metabolic reprogramming to stimulate FAO and OXPHOS pathways. To further examine whether FGK45-induced antitumor responses and anti-tumorigenic polarization in TAMs depend on FAO, lethally irradiated wild-type mice were transplanted with bone marrows from *LysM*-Cre Cas9 mice transduced with lentivirus expressing either control sgRNA or *Cpt1a*-targeting sgRNA. We then engrafted YUMM1.7 melanoma cells into transplanted mice, followed with either PBS or FGK45 treatment (Extended Data Fig. 3f,g). We observed that TAMs from mice reconstituted with *Cpt1a*-deficient bone marrows expressed lower levels of Cpt1a (Extended Data Fig. 3h), and FGK45 treatments failed to suppress tumor growth and tumor burden (Fig. 2g,h). Strikingly, in contrast to TAMs from control mice, FGK45 treatment could not induce anti-tumorigenic and pro-inflammatory genes (Fig. 2i), and repress the pro-tumorigenic genes (Extended Data Fig. 3i) in TAMs from *Cpt1a*-deficient bone marrow-transplanted mice. Collectively, these results demonstrate that CD40 signaling activates a previously unexplored metabolic reprogramming mechanism that exploits fatty acid metabolism to promote macrophage pro-inflammatory and anti-tumorigenic polarization.

### CD40-triggered fatty acid oxidation supports histone acetylation

**Fig. 3:**
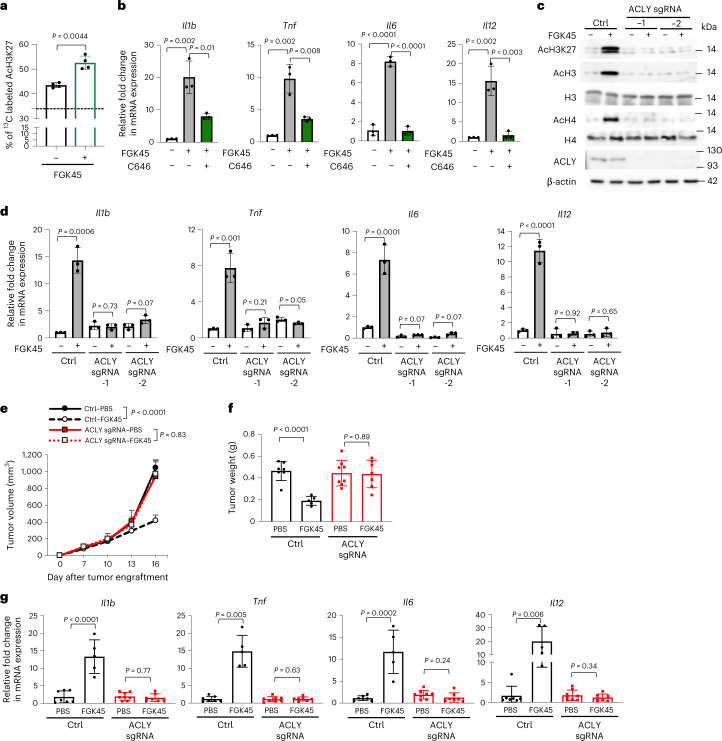
Fatty acid oxidation supports ACLY-mediated histone acetylation. **a**, The percentage of ^13^C-labeled H3K27ac in ^13^C-labeled BMDMs pulsed with palmitic acid stimulated with or without FGK45 mAb was determined by proteomics. The basal labeling level of ^13^C-labeled H3K27ac (dashed line) was determined by the same approach in BMDMs treated with ACLY inhibitor. **b**, qPCR analysis of mRNA expression of pro-inflammatory marker genes in BMDMs cells stimulated with or without FGK45 mAb in the absence or presence of C646, p300/HAT inhibitor, under glucose-deplete media. **c**, Representative immunoblots of indicated proteins in lysates of Cas9-expressing BMDMs harboring control sgRNA (Ctrl) and ACLY gRNAs (ACLY sgRNA-1 and sgRNA-2) stimulated with or without FGK45 mAb under glucose-deplete media. **d**, qPCR analysis of mRNA expression of mRNA expression of pro-inflammatory marker genes in Cas9-expressing BMDMs harboring control sgRNA (Ctrl) and ACLY gRNAs (ACLY sgRNA-1 and sgRNA-2) stimulated with or without FGK45 mAb under glucose-deplete media. **e**,**f**, Tumor growth (**e**) and tumor weight (**f**) in tumor-bearing mice transplanted with either indicated bone marrows and then treated with either PBS or FGK45 mAb. **g**, Relative fold change in mRNA expression of pro-inflammatory marker genes in TAMs isolated from tumor-bearing mice transplanted with either indicated bone marrows and then treated with either PBS or FGK45 mAb. In **a**, representative data or pooled data from two individual experiments with *n* = 3 per group. In **b**–**d**, data are representative of three individual experiments with *n* = 3 per group. **e**–**g**, Representative data or pooled data from two individual experiments with *n* = 4 mice per group in **e**; *n* = 7 for Ctrl-PBS, *n* = 5 for Ctrl-FGK45, *n* = 8 for ACLY gRNA-PBS and *n* = 7 for ACLY gRNA-FGK45 in **f** and *n* = 8 in **g**. All data are the mean ± s.d. and analyzed by a two-tailed Student’s *t*-test. Source data

**Extended Data Fig. 4:**
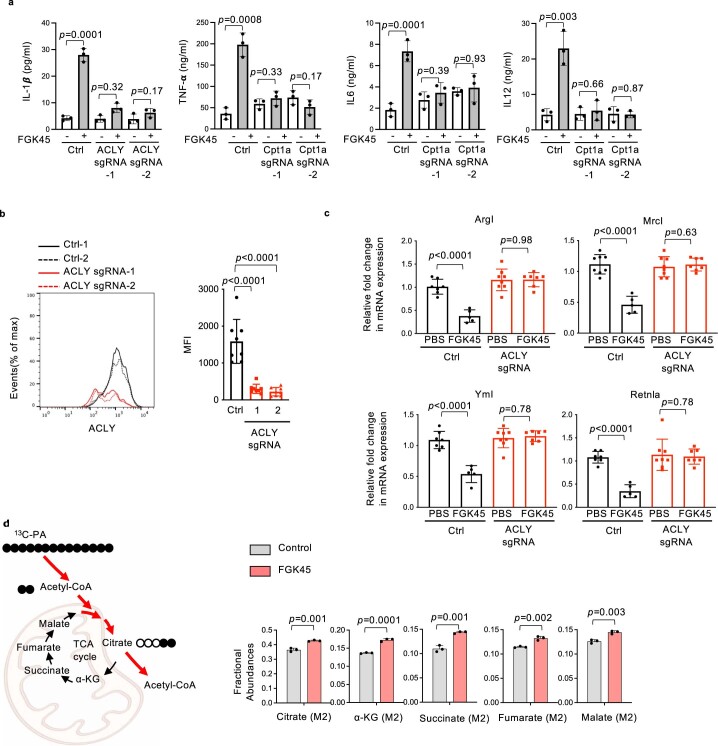
FAO supports CD40-stimulated epigenetic reprogramming. (**a**) Proinflammatory cytokines production in Cas9-expressing BMDMs harboring control sgRNA (Ctrl) and ACLY sgRNAs (ACLY sgRNA-1 and -2) stimulated with or without FGK45 mAb. Representative data from three individual experiments (n = 3 per group). (**b**) Representative flow plot (left) and quantitative results (right) of ACLY expression in TAMs isolated from tumor-bearing mice transplantate with indicated bone marrows. Representative data from two individual experiments and dots represent individual mouse (n = 8 mice per group). (**c**) qPCR analysis of mRNA expression of anti-inflammatory marker genes in TAMs isolated from indicated tumor-bearing treated with control vehicle (PBS) or FGK45 mAb. Representative data or pooled data from two individual experiments (n = 7 for Ctrl-PBS, n = 5 for Ctrl-FGK45, n = 8 for ACLY gRNA-PBS and n = 7 for ACLY gRNA-FGK45). (**d**) Metabolic tracing analysis of 13C-labeled palmitate (PA) in BMDMs stimulated with control vehicle or FGK45 mAb for 3 h. M2 + citrate and other indicated TCA cycle metabolites in the BMDMs of indicated groups were determined by mass spectrometry. Culmulative data of three individual experiments (n = 5 per group). All data are presented as mean ± SD and analyzed by a two-tailed Student’s t-test. Source data

Fatty acids are broken down during FAO to generate acetyl-CoA, which enters the TCA cycle and reacts with oxaloacetate (OAA) to form citrate. Citrate can exit the TCA cycle and, upon ATP citrate lyase (ACLY)-mediated processing, it functions as an acetyl-CoA donor for protein acetylation reactions and epigenetic regulation^25,26^. By tracing palmitate metabolic fate, we found that FGK45 treatment promoted the incorporation of ^13^C carbon from palmitate onto acetylated histone H3 Lys27 (H3K27ac), which represents a relevant epigenetic mark associated to permissive transcriptional states^27^ (Fig. 3a). Moreover, inhibition of histone acetyltransferase with C646 reduced FGK45-mediated induction of pro-inflammatory marker genes (Fig. 3b), suggesting that CD40 signaling promotes pro-inflammatory activation by regulating histone modification with FAO-derived acetyl-CoA. To further investigate this, we applied CRIPSR-based gene ablation in BMDMs as described above. In control BMDMs, FGK45 treatment increased histone H3 (H3ac) and H4 (H4ac) global acetylation states, including H3K27ac. However, ACLY-deficient BMDMs did not show an increase in histone acetylation marks following FGK45 treatment (Fig. 3c). Moreover, ACLY-deficient BMDMs failed to upregulate pro-inflammatory marker genes and cytokine production upon FGK45 stimulation (Fig. 3d and Extended Data Fig. 4a). Next, we reconstituted lethally irradiated wild-type mice with bone marrows from *LysM*-Cre Cas9 mice transduced with lentivirus expressing either control sgRNA or Acly-targeting sgRNA. TAMs from mice reconstituted with ACLY-deficient bone marrows expressed lower levels of ACLY (Extended Data Fig. 4b) and reconstitution with ACLY-deficient bone marrows abolished antitumor responses elicited by FGK45 treatments (Fig. 3e,f). Coherently, FGK45 treatment neither upregulated anti-tumorigenic and pro-inflammatory genes (Fig. 3g), nor repressed the expression of genes related to pro-tumorigenic and tissue-repairing processes (Extended Data Fig. 4c). We next examined the metabolic fate of palmitate by tracing ^13^C-labeled palmitate and found that FGK45 treatment notable increased M2 + citrate as well as other TCA metabolites in BMDMs, highlighting that CD40 signal promotes incorporation of carbons derived from palmitate into citrate. Taken together, these results reveal that the use of fatty acids in response to CD40 signaling supports the epigenetic reprogramming guiding macrophage pro-inflammatory and anti-tumorigenic polarization.

### Lactate production is required for functional reprogramming

**Fig. 4:**
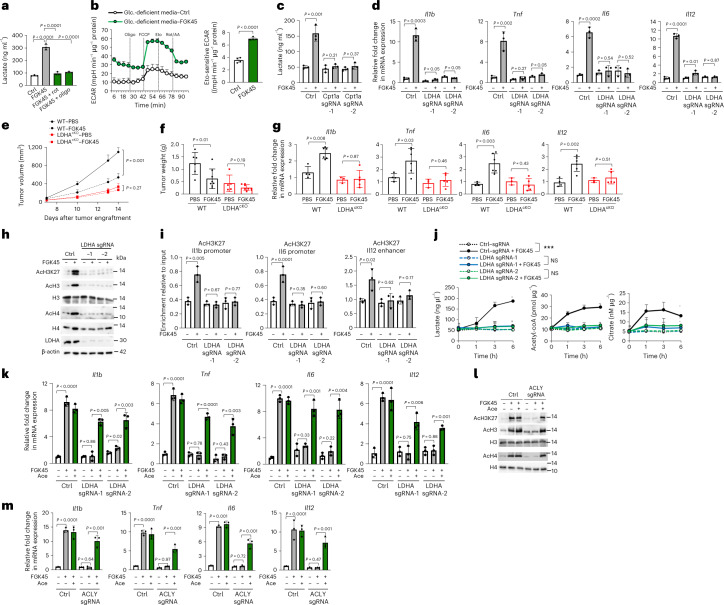
Lactate production is required for epigenetic and functional polarization. **a**, Extracellular lactate production from BMDMs stimulated with control vehicle (Ctrl) or FGK45 mAb in the absence or presence of rotenone or oligomycin under glucose-deplete media. **b**, ECAR measurement (left) and the average of etomoxir (Eto)-sensitive ECAR (right) in BMDMs stimulated with either Ctrl or FGK45 mAb under glucose-deplete media. **c**, Extracellular lactate production from Cas9-expressing BMDMs harboring control sgRNA (Ctrl) and sgRNAs targeting *Cpt1a* (Cpt1a sgRNA-1 and sgRNA-2) under glucose-deplete media. **d**, qPCR analysis of mRNA expression of pro-inflammatory marker genes in BMDMs harboring control sgRNA (Ctrl) and sgRNAs targeting *LdhA* (LDHA sgRNA-1 and sgRNA-2) under glucose-deplete media. **e**,**f**, Tumor growth (**e**) and tumor weight measurement (**f**) in tumor-bearing WT or *LdhA*^cKO^ mice treated with PBS or FGK45 mAb. **g**, qPCR analysis of mRNA expression of pro-inflammatory marker genes in TAM isolated from tumor-bearing WT or *LdhA*^cKO^ mice. **h**, Representative immunoblots of indicated proteins in lysates of Cas9-expressing BMDMs harboring indicated sgRNAs. **i**, Chromatin immunoprecipitation (ChIP) analysis of H3K27ac in Cas9-expressing BMDMs harboring indicated sgRNAs. Data were normalized to input. **j**, The concentration of indicated metabolites in Cas9-expressing BMDMs harboring indicated sgRNAs upon FGK45 stimulation for indicated periods. **k**, qPCR analysis of mRNA expression of pro-inflammatory marker genes in Cas9-expressing BMDMs harboring indicated sgRNAs in the absence or presence of sodium acetate (Ace, 5 mM) under glucose-deplete media. **l**,**m**, Representative immunoblots of indicated proteins (**l**) and qPCR analysis of mRNA expression of pro-inflammatory marker genes (**m**) in of Cas9-expressing BMDMs harboring indicated sgRNAs upon FGK45 mAb under glucose-deplete media with or without acetate (ace). **a**–**d**,**l**,**m**, Data are representative of three individual experiments with *n* = 3 per group. **e**–**g**, Representative data or pooled data from two individual experiments with *n* = 4 mice per group in **e**; *n* = 6 for WT-PBS, *n* = 8 for WT-FGK45, *n* = 5 for LDHA^cKO^-PBS and *n* = 8 for LDHA^cKO^-PBS in **f**; *n* = 4 for WT-PBS, *n* = 6 for WT-FGK45, *n* = 3 for LDHA^cKO^-PBS and *n* = 5 for LDHA^cKO^-PBS in **g**. **h**–**k**, Data are representative of three individual experiments (*n* = 3 per group). All data are the mean ± s.d. and analyzed by a two-tailed Student’s *t*-test. NS, not significant. Source data

**Extended Data Fig. 5:**
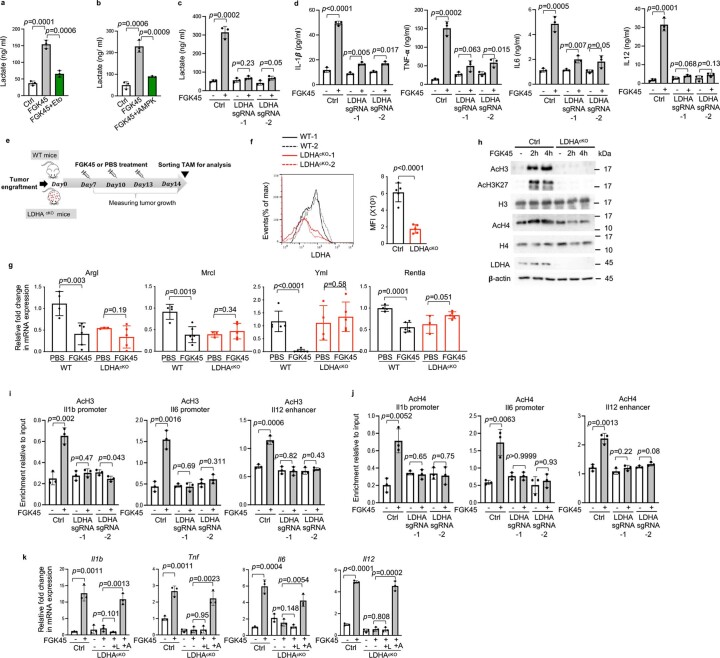
LDHA-dependent lactate production is essential for CD40 signal. (**a–b**) Extracellular lactate production in BMDMs stimulated with or without FGK45 in the absence or presence of etomoxir (Eto) (**a**), or AMPK inhibitor (iAMPK) (**b**) under glucose-deplete media. (**c–d**) Extracellular lactate levels (**c**) and the production of proinflammatory cytokines (**d**) in Cas9-expressing BMDMs harboring control sgRNA (Ctrl) and LGHA sgRNAs (LDHA sgRNA-1 and -2) stimulated with or without FGK45 mAb. (**e**) Illustration of the design for indicated mice follwed with tumor engraftment and then FGK45 treatment. (**f**) Representative flow plot (left) and quantitative results (right) of LDHA expression in TAMs isolated from tumor-bearing mice with indicated genetypes. (**g**) qPCR analysis of mRNA expression of anti-inflammatory marker genes ins TAM isolated from tumor-bearing WT (Ctrl) and *LdhA*^cKO^ mice. (**h**) Representative immunoblots of indicated proteins in wild type BMDMs (Ctrl) and LDHA-KO BMDM (LDHA^cKO^) stimulated with or without FGK45 mAb for ndicated periods. (**i–j**) Chromatin-immunoprecipitation (ChIP) analysis of acetylated histone H3 (AcH3) (**i**) or acetylated histone H4 (AcH4) (**j**) in Cas9-expressing BMDMs harboring control sgRNA (Ctrl) and *LdhA* gRNAs (LDHA sgRNA-1 and -2) with or without FGK45 mAb stimulation after real-time qPCR of enhancer/ promoter reguons on the proinflammatory marker gene loci. Data are normalized to input. (**k**) qPCR analysis of mRNA expression of proinflammatory marker genes in wld type BMDMs (Ctrl) and LDHA-KO BMDMs (LDHA^cKO^) stimulated with or without FGK45 mAb in the absence or presence of lactate (L; 10 mM) or sodium acetate (A; 5 mM) under glucose-deplete media. (**a–d**, **i–k**) Representative data from three individual experiments (n = 3 per group). (**e–g**) Representative pooled data from two individual experiments with n = 5 mice per group in (**f**) and n = 6 for WT-PBS, n = 8 for WT-FGK45, n = 5 for LDHA^cKO^-PBS and n = 8 for LDHA^cKO^-PBS in (**g**). All data are presented as mean ± SD and analyzed by a two-tailed Student’s t-test. Source data

Our above findings indicate that, FAO can be both implicated in the promotion of anti-inflammatory polarization^4,9,10^, and in the acquisition of the pro-inflammatory and anti-tumorigenic polarization state in macrophages. Notably, we also observed that, in contrast to LPS, CD40 signaling regulates ECAR and lactate production even in macrophages under glucose-limited conditions (0.1 mM glucose). However, it remains unclear whether the production of lactate is essential for pro-inflammatory polarization. We then examined whether OXPHOS and FAO are needed for FGK45-induced lactate production. OXPHOS inhibitors, including oligomycin and rotenone, blocked FGK45-induced lactate production (Fig. 4a) and etomoxir suppressed FGK45-induced ECAR and lactate production (Fig. 4b and Extended Data Fig. 5a). Consistently, Cpt1a-deficient BMDMs failed to induce lactate production upon FGK45 treatment (Fig. 4c). In addition, inhibition of AMPK with dorsomorphin prevented FGK45-triggered lactate production (Extended Data Fig. 5b). These results imply that lactate production is linked to the metabolic reprogramming and signaling cascades driving CD40-mediated macrophage polarization. To further examine whether lactate production is needed for CD40-mediated macrophage polarization, we ablated the *Ldha* gene, encoding lactate dehydrogenase A (LdhA), a rate-limiting enzyme for lactate production by expressing an LDHA-targeting sgRNA in BMDMs derived from *LysM*-Cre Cas9 mice. LdhA-deficient BMDMs produced less lactate upon FGK45 treatments (Extended Data Fig. 5c) and failed to induce expression of pro-inflammatory marker genes and production of cytokines (Fig. 4d and Extended Data Fig. 5d), indicating that LdhA-dependent lactate production is an essential component for CD40-driven macrophage polarization. Next, we treated tumor-bearing wild-type mice or *LysM*-Cre *LdhA*^fl/fl^ mice (referred to as *Ldha* conditional knockout mice, *LdhA*^cKO^) with FGK45 monoclonal antibodies and found that TAMs from *LdhA*^cKO^ mice displayed reduced expression of LdhA compared to TAMs from wild-type mice (Extended Data Fig. 5e,f). Furthermore, FGK45 treatments did not suppress tumor growth in *LdhA*^cKO^ mice (Fig. 4e,f). Consistently, TAMs from *LdhA*^cKO^ mice did not upregulate expression of anti-tumorigenic genes (Fig. 4g) and failed to suppress expression of pro-tumorigenic genes upon FGK45 treatments (Extended Data Fig. 5g). These results imply that glucose-independent, FAO-dependent lactate production is critical for CD40-driven macrophage polarization.

Next, we found that BMDMs from both *LdhA*^cko^ mice and LysM-Cre Cas9 mice expressing *Ldha*-taregting sgRNAs, could not increase the global acetylation state of histone H3 and H4 upon FGK45 treatment (Fig. 4h and Extended Data Fig. 5h). Furthermore, FGK45 could not increase active epigenetic marks at promoters and enhancers of pro-inflammatory marker genes *Il1b*, *Il6* and *Il12b* (Fig. 4i and Extended Data Fig. 5i,j), indicating that LdhA-mediated lactate production is required for CD40-driven epigenetic reprogramming. Next, we found that FGK45 stimulation increased citrate and acetyl-CoA levels in control BMDMs, but not in LdhA-deficient BMDMs (Fig. 4j), indicating that LdhA controls the production of citrate and acetyl-CoA, which can be used for epigenetic reprogramming. In support of this, acetate supplementation, which replenishes acetyl-CoA levels^28^, restored CD40-triggered expression of pro-inflammatory marker genes in LdhA-deficient BMDMs (Fig. 4k). It is noteworthy that lactate supplementation failed to restore FGK45-induced expression of pro-inflammatory marker genes in BMDMs from *LdhA*^cKO^ mice (Extended Data Fig. 5k), suggesting that lactate production regulates CD40-orchestrated macrophage polarization by facilitating the production of acetyl-CoA, rather than through other lactate-specific functions. Moreover, we found that supplementation of acetate could restore FGK45-induced changes in acetylated histone state and pro-inflammatory gene expression in ACLY-deficient BMDMs (Fig. 4l,m). Altogether, our results show that CD40 signaling stimulates glucose-independent lactate production to drive epigenetic and functional reprogramming of macrophages.

### Lactate production supports metabolic reprogramming

**Fig. 5:**
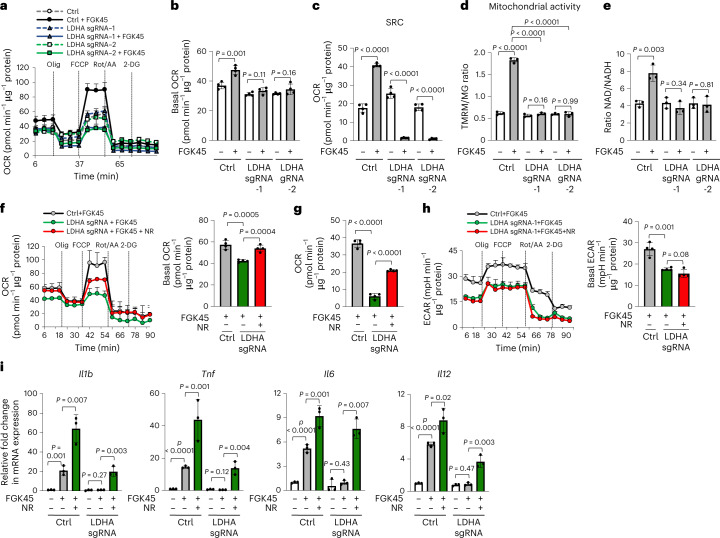
Lactate production supports OXPHOS by tailoring NAD^+^-to-NADH balance. **a**–**c**, OCR measurement (**a**), the average of basal OCR (**b**) and SRC (**c**) in Cas9-expressing BMDMs harboring control sgRNA (Ctrl) and LDHA gRNAs (LDHA sgRNA-1 and sgRNA-2) stimulated with either Ctrl or FGK45 mAb under glucose-deplete media following the additions of oligomycin, FCCP, Rot/AA and 2-deoxyglucose (2-DG). **d**,**e**, The ratios of TMRM to MG (**d**) and the ratios of NAD^+^ to NADH (**e**) in Cas9-expressing BMDMs harboring control sgRNA (Ctrl) and LDHA gRNAs (LDHA sgRNA-1 and sgRNA-2) stimulated with either Ctrl or FGK45 mAb under glucose-deplete media. **f**,**g**, The real-time change of OCR (**f**, left), the average of basal OCR (**f**, right) and SRC (**g**) in Cas9-expressing BMDMs harboring control sgRNA (Ctrl) and LDHA gRNAs (LDHA sgRNA-1 and sgRNA-2) stimulated with either control vehicle (Ctrl) or FGK45 mAb in the absence or presence of NR under glucose-deplete media. **h**, The real-time change of ECAR (left) and the average of basal ECAR (right) in Cas9-expressing BMDMs harboring control sgRNA (Ctrl) and LDHA gRNAs (LDHA sgRNA-1 and sgRNA-2) stimulated with either control vehicle (Ctrl) or FGK45 mAb in the absence or presence of NR under glucose-deplete media. **i**, qPCR analysis of mRNA expression of pro-inflammatory marker genes in Cas9-expressing BMDMs harboring control sgRNA (Ctrl) and LDHA gRNA (LDHA sgRNA) stimulated with or without FGK45 in the absence or presence of NR. Data are representative of three individual experiments with *n* = 3 per group in **a**–**c** and **f**–**h** and *n* = 3 per group in **d**, **e** and **i**. All data are the mean ± s.d. and analyzed by a two-tailed Student’s *t*-test. Source data

Our results show that LDHA prompts pro-inflammatory/anti-tumorigenic activation in macrophages by controlling the production of citrate and acetyl-CoA. However, it remains unclear how LDHA-mediated lactate production regulates CD40-driven metabolic reprogramming. We examined the metabolic profiles of FGK45-treated BMDMs and found that LdhA-deficient BMDMs failed to increase basal OCR and SRC upon FGK45 treatments (Fig. 5a–c). Moreover, the disruption of LdhA prevented CD40-induced mitochondrial activity (Fig. 5d), suggesting that lactate production is required for CD40-mediated regulation of OXPHOS. Notably, FGK45 treatment increased the NAD^+^-to-NADH ratio in control, but not LdhA-deficient, BMDMs (Fig. 5e). Interestingly, lactate production is known to increase NAD^+^-to-NADH ratios that further support NADH-producing metabolic processes, such as glycolysis^9^. Thus, it is likely that the reduced NAD^+^-to-NADH ratio in LdhA-deficient macrophages leads to their inability to engage in OXPHOS upon FGK45 stimulation. Next, we tested whether boosting the NAD^+^-to-NADH ratio could restore CD40-driven OXPHOS and pro-inflammatory polarization in LdhA-deficient macrophages. Our results showed that nicotinamide riboside (NR) supplementation to replenish NAD^+^ (refs. ^29,30^) restored basal OCR and SRC, but did not affect ECAR, in LdhA-deficient BMDMs (Fig. 5f–h). Intriguingly, NR supplementation partially restored the expression of pro-inflammatory marker genes in BMDMs treated with FGK45 monoclonal antibodies, even though ECAR was unaffected (Fig. 5i). Taken together, our results show that CD40-induced lactate production fine-tunes OXPHOS by adjusting the NAD^+^-to-NADH ratio that promotes epigenetic and functional reprogramming for pro-inflammatory macrophage polarization.

### Glutamine metabolism feeds lactate production

**Fig. 6:**
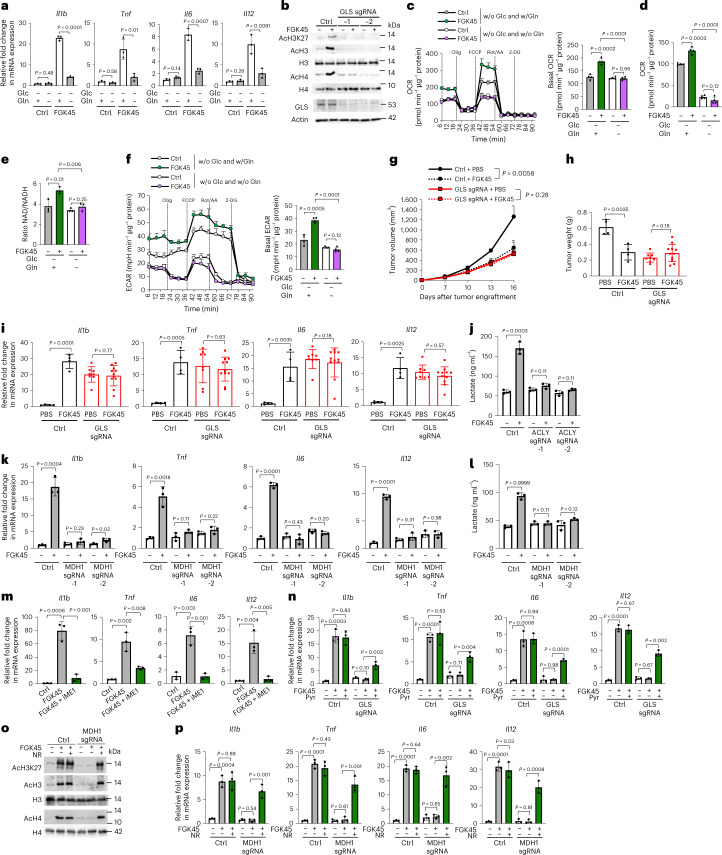
Glutamine metabolism feeds CD40-induced lactate production. **a**, mRNA expression of pro-inflammatory marker genes in BMDMs stimulated with Ctrl or FGK45 mAb under glucose (Glc) or glutamine (Glu) -replete or -deplete conditions. **b**, Representative immunoblots of indicated proteins in Cas9-expressing BMDMs harboring indicated sgRNAs upon FGK45 mAb treatment under glucose-deplete media. **c**,**d**, The change of OCR (**c**, left), the average of basal OCR (**c**, right) and SRC (**d**) in BMDMs stimulated with either Ctrl or FGK45 mAb under glucose (Glc) or glutamine (Glu) -replete or -deplete conditions. **e**,**f**, The ratios of NAD^+^ to NADH (**e**), the change of ECAR (**f**, left) and the average of basal ECAR (**f**, right) in BMDMs stimulated with either Ctrl or FGK45 mAb in indicated conditions. **g**,**h**, Tumor growth (**g**) and tumor weight (**h**) in tumor-bearing mice transplanted with either indicated bone marrows under indicated treatments. **i**, Relative fold change in mRNA expression of pro-inflammatory marker genes in TAMs isolated from indicated tumor-bearing mice. **j**, Extracellular lactate production from Cas9-expressing BMDMs harboring indicated sgRNAs upon FGK45 treatment. **k**,**l**, qPCR analysis of mRNA expression of pro-inflammatory marker genes (**k**) and extracellular lactate production (**l**) in BMDMs harboring indicated sgRNAs upon stimulation. **m**, qPCR analysis of mRNA expression of pro-inflammatory marker genes in BMDMs stimulated in the absence or presence of malic enzyme inhibitor (ME1). **n**, mRNA expression of pro-inflammatory marker genes in Cas9-expressing BMDMs harboring indicated sgRNAs in the absence or presence of sodium pyruvate (Pyr). **o**,**p**, Representative immunoblots of indicated proteins (**o**) and mRNA expression of pro-inflammatory marker genes (**p**) in Cas9-expressing BMDMs harboring indicated sgRNAs stimulated with or without FGK45 mAb with or without NR. Data are representative of three independent experiments with *n* = 3 per group in **a**, **b** and **j**–**p** and *n* = 4 per group in **c**, **d** and **f**. **g**,**i**,**o**,**p**, Representative data or pooled data from two individual experiments with *n* = 4 mice per group in **g**; *n* = 4 for Ctrl-PBS, *n* = 4 for Ctrl-FGK45, *n* = 8 for GLS gRNA-PBS and *n* = 11 for GLS gRNA-FGK45 in **h** and *n* = 8 (**i**). All data are the mean ± s.d. and analyzed by a two-tailed Student’s *t*-test. Source data

**Extended Data Fig. 6:**
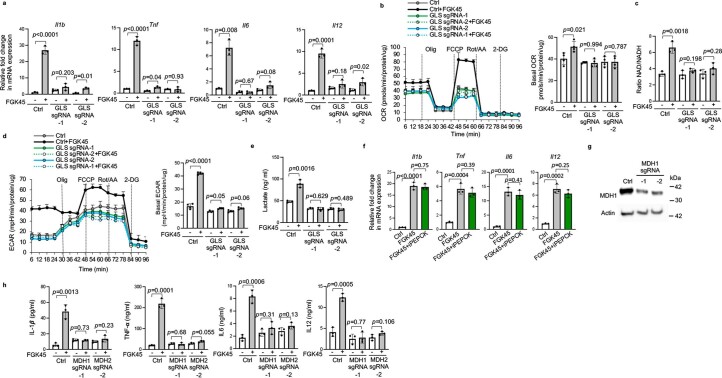
Glutamine metabolism supports CD40-mediated macrophage polarization. (**a**) qPCR analysis of mRNA expression of proinflammatory marker genes in Cas9-expressing BMDMs harboring control sgRNA (Ctrl) and GLS gRNAs (GLS sgRNA-1 and -2) with or without FGK45 mAb stimulation. (**b–c**) OCR measurement (**b**; left), the average of basal OCR (**b**; right), and NAD^+^-TO-NADH ratios (**c**) in Cas9-expressing BMDMs harboring control sgRNA (Ctrl) and GLS gRNAs (GLS sgRNA-1 and -2) stimulated with or without FGK45 mAb under glucose-deplete media. (**d**, **e**) ECAR measurement (**d**; left), the average of basal ECAR (**d**; right), and extracellular lactate production (**e**) in Cas9-expressing BMDMs harboring control sgRNA (Ctrl) and GLS gRNAs (GLS sgRNA-1 and -2) stimulated with or without FGK45 mAb under glucose-deplete media. (**f**) qPCR analysis of mRNA expression of proinflammatory marker genes in BMDMs stimulated with or without FGK45 mAb in the absence or presence of PEPCK inhibitor (iPEPCK) under glucose-deplete media. (**g**) Representative immunoblots of MDH1 and β-actin in Cas9-expressing BMDMs harboring control sgRNA (Ctrl) and MDH1 gRNAs (MDH1 sgRNA-1 and -2). (**h**) qPCR analysis of mRNA expression of proinflammatory marker genes in Cas9-expressing BMDMs harboring control sgRNA (Ctrl) and MDH1 gRNAs (MDH1 sgRNA-1 and -2) stimulated with or without FGK45 mAb. (**a–h**) Representative data from three individual experiments (n = 3 per group in **a, e, f, h**; n = 4 per group in b-d). All data are presented as mean ± SD and analyzed by a two-tailed Student’s t-test. Source data

We observed that FGK45 treatment can stimulate lactate production in the absence of glucose, suggesting the requirement of alternative nutrients for CD40 signaling-mediated macrophage polarization. Interestingly, glutamine, like glucose, is metabolized into lactate in tumor cells^31^. However, glutamine metabolism is known to support anti-inflammatory polarization^5,24^ and it remains to be established whether immune cells can convert glutamine into lactate. We first found that glutamine deprivation rather than glucose depletion impaired FGK45-induced expression of pro-inflammatory marker genes (Fig. 6a). Moreover, BMDMs expressing sgRNA targeting the *Gls* gene, encoding glutaminase 1, were unable to induce epigenetic modifications, including H3K27ac, H3ac and H4ac (Fig. 6b), and failed to upregulate pro-inflammatory polarization upon FGK45 stimulation (Extended Data Fig. 6a). In addition, glutamine deprivation and Gls1 deficiency led to a reduction in OCR and SRC in response to FGK45 treatment (Fig. 6c,d and Extended Data Fig. 6b). Intriguingly, both glutamine deprivation and Gls1 deficiency abolished FGK45-mediated induction of the NAD^+^-to-NADH ratio in BMDMs (Fig. 6e and Extended Data Fig. 6c), suggesting that glutamine metabolism may control lactate production, which in turn regulates NAD^+^-to-NADH ratios. Indeed, glutamine deprivation and GLS1 deficiency abolished FGK45-stimulated ECAR and lactate production (Fig. 6f and Extended Data Fig. 6d,e). We further examined whether FGK45-induced antitumor responses and anti-tumorigenic polarization of TAMs rely on glutamine metabolism. Immunologic reconstitution of Gls1-deficient bone marrows abolished FGK45-elicited antitumor responses and impaired the induction of genes related to anti-tumorigenic functions in TAMs from mice transplanted with Gls1-deficient bone marrow (Fig. 6g–i).

**Extended Data Fig. 7:**
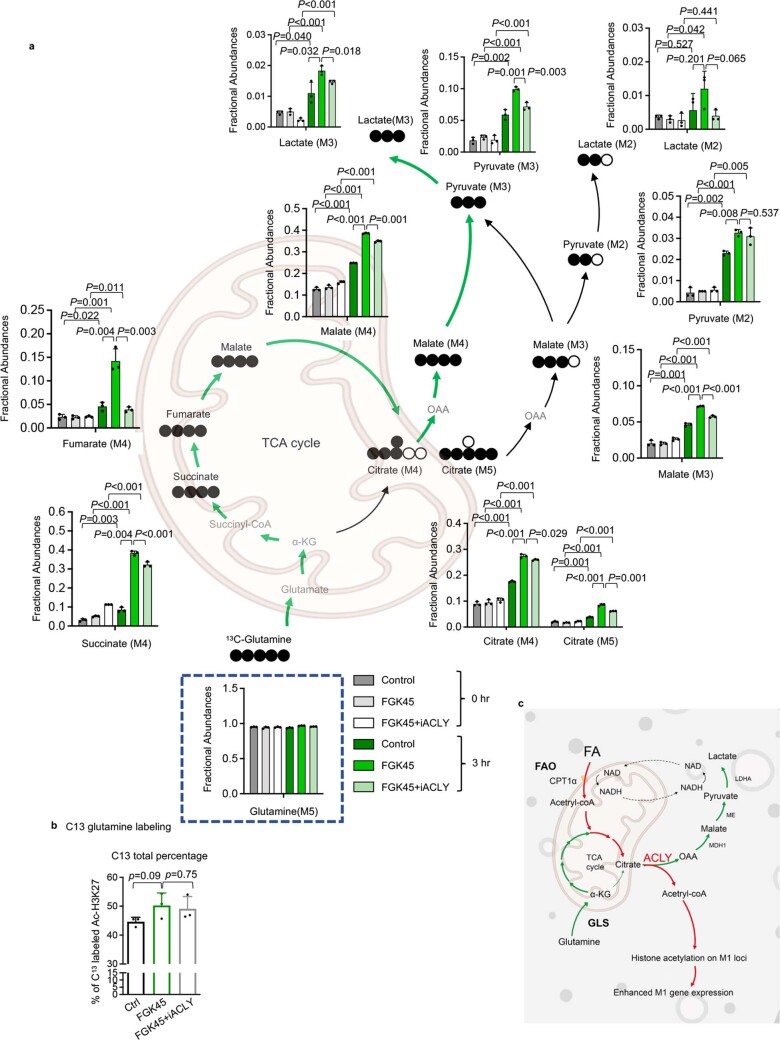
Metabolic fate of glutamine upon CD40 stimulation. (**a**) Metabolic tracing analysis of 13C-labeled glutamine in BMDMs stimulated with control vehicle or FGK45 mAb for 0 h or 3 h. Indicated labeled metabolites in the BMDMs of indicated groups were determined by mass spectrometry. (**b**) The percentage of ^13^C labeled acetylated histone H3-K27 (Ac-H3K27) in ^13^C-labeled glutamine pulsed BMDMs stimulated with or without FGK45 mAb was determined by proteomics. The basal labeling level of ^13^C labeled acetylated histone H3-K27 was determined by the same approach in BMDMs treated with FGK45 mAb plus ACLY inhibitor. Data are presented as mean ± SD. (**c**) Illustration of metabolic reprogramming induced by CD40 signal in macrophages. Representative data from two individual experiments (n = 3 per group). All data are presented as mean ± SD and analyzed by a two-tailed Student’s t-test. Source data

Glutamine can be metabolized into alpha-ketoglutarate, which enters the TCA cycle. In the TCA cycle, OAA can be converted by phosphoenolpyruvate carboxykinase (PEPCK) into phosphoenolpyruvate, a glycolytic metabolite for gluconeogenesis and pyruvate production^32^. However, 3-mercaptopicolinic acid, an inhibitor of PEPCK, did not affect the expression of pro-inflammatory marker genes upon FGK45 stimulation (Extended Data Fig. 6f). Unexpectedly, lactate production did not increase in ACLY-deficient BMDMs following FGK45 treatment (Fig. 6j). ACLY breaks down citrate into acetyl-CoA and OAA^25^; the latter can be further transformed into pyruvate through a series of reactions controlled by cytosolic enzymes malate dehydrogenase 1 (MDH1) and malic enzyme (ME1)^33,34^. Therefore, we hypothesized that the conversion of glutamine into lactate occurs via the production of pyruvate as a metabolic intermediate through the coordinated action of ACLY, MDH1 and ME1 enzymes. We then genetically ablated the *Mdh1* gene by transducing BMDMs from *LysM*-Cre Cas9 mice with lentiviruses expressing an sgRNA targeting *Mdh1* (Extended Data Fig. 6g). We found that FGK45 treatment failed to induce the expression of pro-inflammatory marker genes and the production of cytokines in Mdh11-deficient BMDMs (Fig. 6k and Extended Data Fig. 6h). In addition, loss of MDH1 activity prevented CD40-stimulated lactate production (Fig. 6l), indicating that MDH1-mediated OAA-to-malate conversion is required for lactate production and pro-inflammatory polarization upon CD40 signaling activation. Moreover, targeting ME1 to prevent malate-to-pyruvate conversion dramatically impaired CD40-driven pro-inflammatory polarization (Fig. 6m). Intriguingly, we found that supplementation of sodium pyruvate could restore FGK45-induced pro-inflammatory polarization in Gls1-deficient BMDMs (Fig. 6n). Of note, NR supplementation could restore acetylated histone state and the induction of pro-inflammatory gene expression in MDH1-deficient BMDMs stimulated with FGK45 monoclonal antibody (Fig. 6o,p), which further strengthens our model that Mdh1-supported lactate production plays a critical role for ensuring CD40 signal-induced pro-inflammatory activation by tailoring NAD-to-NADH balance. We then traced the metabolic fate of ^13^C-labeled glutamine upon FGK45 treatment. Our result showed that FGK45 treatment robustly increased the accumulation of labeled succinate (M4 labeled), fumarate (M4 labeled), pyruvate (M3 labeled) and lactate (M3 labeled) in an ACLY inhibitor-sensitive manner (Extended Data Fig. 7a), supporting our model that glutamine is used for lactate production. Of note, we found that the levels of M5 + citrate were lower than M4 + citrate, suggesting that reductive glutamine carboxylation can only be slightly engaged in FGK45-stimulated BMDMs. Furthermore, we followed glutamine metabolic fate with isotope tracing and observed that FGK45 treatment did not promote the incorporation of ^13^C carbon from glutamine onto H3K27ac (Extended Data Fig. 7b), highlighting that glutamine does not provide acetyl-CoA for histone acetylation through reductive glutamine carboxylation. Taken together, these results demonstrate that CD40 signaling controls a new metabolic network involving ACLY, MDH1 and ME1 that shunts glutamine into lactate to drive macrophage polarization rather than using glutamine to modulate acetylated histone state.

## Discussion

Our study reveals that CD40-mediated signaling drives pro-inflammatory/anti-tumorigenic polarization in macrophages by rewiring fatty acid and glutamine metabolic pathways (Extended Data Fig. 7c). In contrast with previous observations linking FAO and glutamine metabolism to anti-inflammatory activation in macrophages^4,24^, here we show that CD40 signaling can coordinate these metabolic processes to orchestrate the epigenetic reprogramming underlying pro-inflammatory/anti-tumorigenic polarization. Furthermore, we reveal that glutamine metabolism feeds the production of lactate that, in turn, sustains FAO by fine-tuning NAD^+^-to-NADH ratio. Lastly, our study demonstrates that some metabolic interventions, including the targeting of LdhA and glutamine metabolism, abolish antitumor responses induced by the agonistic CD40 monoclonal antibodies, suggesting that stronger antitumor responses could be elicited by using CD40 monoclonal antibodies when we could precondition the metabolic milieu of TME.

In contrast to LPS-mediated M1 activation, which is highly sensitive to glucose deprivation^9–11^, we show that CD40 signaling drives M1 activation via a glucose-independent metabolic reprogramming. CD40-induced M1 activation allows glutamine metabolism to feed lactate production, implying that CD40 signaling does not result in a broken TCA cycle in contrast to LPS stimulation. Emerging evidence supports the idea that LPS-induced, autocrine type I interferon leads to TCA cycle disruption as a result of increased *Acod1* expression and itaconate production^35^. Interestingly, macrophages stimulated with agonistic CD40 monoclonal antibodies do not produce type I interferon and show a low level of type I interferon gene signature^16,17^. Therefore, we speculate that the absence of type I interferon production in response to CD40 signaling activation allows macrophages to maintain the metabolic flexibility required to use glutamine and fatty acids while preserving an intact TCA cycle. Intriguingly, instead of supporting M2 polarization, we show that glutamine metabolism and FAO can promote M1 polarization and anti-tumorigenic activation of macrophages following anti-CD40 monoclonal antibodies treatment. Moreover, ACLY, which was previously linked to macrophage polarization^26,36,37^, is also required for the epigenetic reprogramming underlying CD40-mediated M1 activation. Therefore, it is likely that metabolic flexibility and ACLY-dependent activity together with key pro-inflammatory signaling pathways downstream of CD40, such as nuclear factor-kappa B, promote pro-inflammatory/anti-tumorigenic polarization rather than M2 polarization. It would be interesting to explore: (1) how pro-inflammatory and anti-inflammatory signaling cascades integrate with the metabolic reprogramming to instruct macrophage polarization; and (2) how metabolic flexibility can be exploited to reeducate macrophages for disease treatments. Answering these questions would provide the foundation for the development of novel therapeutical strategies aimed at tailoring macrophage functions.

The metabolic fate of the OAA produced by ACLY is less clear compared to that of acetyl-CoA, which can be used for protein modifications and lipogenesis^25,38^. Interestingly, ALCY-generated OAA was shown to re-enter the TCA cycle through a non-canonical cycle configuration, which is required for stem cell fate transitions^39,40^. In this study, we show that OAA produced by ACLY can be also metabolized into lactate through the coordinated activity of cytosolic MDH1 and ME1. This non-canonical conversion of OAA to lactate fine-tunes the NAD^+^-to-NADH ratio to sustain the metabolic programs, including FAO and OXPHOS, required for the CD40-induced pro-inflammatory polarization. ACLY-mediated OAA-to-lactate conversion has been reported to support hippocampus metabolic fitness in response to limited glucose availability^41^. Interestingly, the conversion of citrate-derived OAA into pyruvate was shown to support NADPH production, which can be further used for nitric oxide generation^42^. Therefore, the OAA-to-lactate conversion identified in this study might provide macrophages with the metabolic adaptation to support their functional polarization in a glucose-limited environment.

The activation of APCs and reprogramming of the immune state in the TME with agonistic CD40 monoclonal antibodies represent a promising therapeutical strategy for the treatment of patients with cancer and the activation of APCs can further support T cell-based antitumor immunity. The concept of combining agonistic monoclonal antibody treatment with other interventions, including radiotherapy, chemotherapy and drugs targeting cancer and immune metabolism is surfacing^13^. Here, we demonstrate that the inhibition of LDHA and GLS1—two metabolic targets that are currently under investigations in clinical trials for cancer therapy—impairs anti-CD40 monoclonal antibody-induced antitumor responses. The therapeutical targeting of cancer metabolism as well as of the metabolic cross-talk between cancer and infiltrating immune cells is believed to support host antitumor immunity. Therefore, future investigations should address whether preconditioning the tumor metabolic milieu with treatments favoring metabolic reprogramming, such as those identified in our study, can potentiate agonistic anti-CD40-mediated antitumor responses. Results from such investigations will provide the foundation for the development of combination therapies using agonist anti-CD40 monoclonal antibodies in cancer treatment.

## Methods

### Mice

C57BL/6/J mice, *Ldha*^fl/fl^ mice, *LysM*-Cre mice and Rosa26-Cas9 knockin mice were purchased from The Jackson Laboratory. *LdhA*^cKO^ mice were bred and maintained at the animal facility of the University of Lausanne, Switzerland. *LysM*-Cre Cas9 mice and mice receiving bone marrow transplantation were bred and maintained at the National Health Research Institutes laboratory animal center. All animal experiments were approved and performed in accordance with guidelines and regulations of the Institutional Animal Care and Use Committees of National Health Research Institutes laboratory animal center with a maximal tumor size < 2.5 cm^3^ or Swiss federal regulations and approved by the veterinary authority of Canton Vaud with a maximal tumor size < 1 cm (ref. ^3^). Both genders of mouse strains were used and mice within 5–10 weeks old were used.

### Bone marrow transplantation

The macrophages-specific gene targeting is established by the CRISPR–Cas9 technology. Donor bone marrow cells were isolated from femurs and tibias of *LysM*-Cre Cas9 mice and transduced with lentivirus expressing mCherry fluorescence and sgRNA targeting indicated genes or a scramble sequence. Transduced bone marrow cells were cultured in the presence of puromycin for 3 d before FACS sorting to enrich mCherry^+^ donor bone marrow cells. Recipient mice were exposed with a single whole-body irradiation at a dose of 8 Gy using the Rad Source RS‐2000 X‐Ray irradiator. After the irradiation, each recipient mouse was injected with 5 × 10^6^ mCherry^+^ donor bone marrow cells via intravenous injection. Recipient mice were allowed 4 weeks to reconstitute their hematopoietic systems before tumor challenge.

### Bone marrow-derived macrophage generation and FGK45 stimulation

Bone marrow cells were collected from femurs and tibias of mice and cultured in DMEM (Thermo Fisher) supplemented with 10% FBS (Thermo Fisher), 1% penicillin–streptomycin (Life Technologies) and 15% L929 cell culture supernatant for BMDMs differentiation for 7 d. On day 7, differentiated BMDMs were re-plated with DMEM (without L929 cell culture supernatant) overnight and plated for experiments. For in vitro FGK45 stimulation, FGK45 (Bio X Cell, BP0016-2, anti-mouse CD40 monoclonal antibody, 20 ng ml^−1^) was crosslinked with goat anti-rat immunoglobulin G (10 ng ml^−1^; BioLegend, 405401) for 30 min at room temperature before being added to the DMEM culture medium. For specific culture condition, BMDM cells were incubated with glutamine and pyruvate-free DMEM medium (Gibco, A1443001) with dialyzed FBS (Thermo Fisher 26400044) plus 0.1 mM glucose for further experiments. In some experiments, BMDMs were treated with FGK45 in the presence of inhibitors were used as follows: etomoxir, 200 μM (Sigma); ACLY inhibitor SB-204990, 40 μM (Sigma); p300 inhibitor C646, 10 μM; AMPK inhibitor dorsomorphin, 20 μM (Kayman no. 11967); Malic enzyme inhibitor (ME1), 1 μM; PEPCK inhibitor, 5 μM; NR, 0.5 mM) in specific culture media as indicated in the figures.

### Generation of bone marrow-derived macrophages with gene ablation

sgRNAs targeting genes of interest were designed and selected from the CRISPRdirect (https://crispr.dbcls.jp/) and CRISPR design (https://www.benchling.com/) tools. All the chosen sgRNAs (Supplementary Table 1) were cloned into pU6-sgRNA-PeGFP-I2-puro vector, and verified by sequencing using the human U6 sequencing primer. For generation of sgRNAs expressing lentivirus, 293T cells were co-transfected with pU6-sgRNA. pU6-sgRNA-PeGFP-I2-puro vector, psPAX2 (Addgene, 12260) and pMD2.G (Addgene, 12259) using Lipofectamine LTX Reagent (Thermo Fisher) and OptiMEM medium (Thermo Fisher, 11058021) according to the manufacturer’s guidelines. To obtain the target gene-knockout BMDMs, bone marrow cells isolated from LysM-Cre Cas9 mice were cultured in the presence of polybrene (8 mg ml^−1^) and sgRNA-expressing lentivirus. Transduced bone marrow cells were cultured with DMEM supplemented with 10% FBS and 20% L929 cell culture supernatant for BMDM differentiation. To generate optimal knockout BMDMs, day 3 after transduction, 15 µg ml^−1^ puromycin (Thermo Fisher) was added. On day 8, the transduced BMDMs were collected by FACS sorter based on mCherry expression and re-plated with DMEM (without L929 cell culture supernatant) overnight for in vitro experiments.

### RNA purification, RT–PCR and qPCR

Total RNA extraction from cells were isolated with TRIzol reagent (Life Technologies) or the miRNeasy Mini kit (Qiagen). RNA was converted into cDNA using M-MLV Reverse Transcriptase (Promega). Quantitative real-time PCR was performed in triplicate on a LightCycler 480 Instrument II machine (Roche Life Science) using the KAPA SYBR FAST qPCR Kit Master Mix (KAPA Biosystems) for quantification of the target gene expression. Relative expression of target genes was normalized to β-actin for each sample. qPCR primer sequences are list in Supplementary Table 2.

### Chromatin immunoprecipitation

ChIP was performed using the ChIP Assay Kit (17–295, Sigma-Aldrich) according to the manufacturer’s instructions. Briefly, 2 × 10^7^ BMDMs were fixed with 10% formaldehyde to cross-link histones to DNA. Cells were lysed with hypotonic buffer (0.3% NP40, 0.1 mM EDTA, 10 mM HEPES (pH 7.9), 10 mM KCl) to enrich nuclei. Chromatin was sheared by micrococcal nuclease (Mnase; NEB (Bioconcept); M0247S) for 10 min at 37 °C, and the reactions were stopped by addition of lysis buffer (1% Triton X-100, 1 mM EDTA, 50 mM HEPES (pH 7.9), 0.4 M NaCl and proteinase inhibitor cocktail). The soluble chromatin supernatant was immunoprecipitated with anti-acetyl-histone H3 (Millipore, 06-599), anti-acetyl-histone H4 (Millipore, 06-866) and anti-histone H3 (acetyl K27) antibody (Abcam, 4729) or IgG (Santa Cruz, SC-2027) antibodies. Immunoprecipitated DNA and input DNA were analyzed by RT–qPCR, and results are presented as a percentage of input. The primers were used for amplification of promoters and enhancer of M1 gene are summarized in Supplementary Table 3.

### Tumor digestion and flow cytometry

Tumor samples were processed to produce a single-cell suspension as previously described^20^. Leukocytes were resuspended in FACS buffer with 5 μg ml^−1^ αCD16/CD32 (2.4G2; BioLegend) for blocking Fc receptors and then first stained with LIVE/DEAD Fixable Violet Cell Stain Kit (Thermo Fisher) for 5 min at room temperature before staining for specified surface markers at 4 °C for 30 min as described previously^29^. For detection of intracellular molecules, the BD Cytofix/Cytoperm Fixation/Permeabilization Kit (554714) was used according to the manufacturer’s protocol and then stained with the relevant antibodies. The following antibodies were used from BioLegend: Brilliant Violet 650 anti-mouse CD45 (30-F11; 03151; 1:500 dilution), Pacific blue anti-mouse/human CD11b (M1/70; 101224; 1:500 dilution), PE/Cy7 anti-mouse Ly-6G/Ly-6C (Gr-1; RB6-8C5; 108416; 1:200 dilution) and APC-C7 anti-mouse F4/80 (BM8; 123116; 1:100 dilution). Sorting of TAMs (CD45^+^Cd11b^+^Gr1^−^F4/80^+^) from tumors was performed using an Influx (BD Biosciences) with a 140-μm nozzle and 7.5 psi of pressure, to a purity of ~95–99%. Data were acquired on an Attune NxT Flow Cytometer (Thermo Fisher Scientific) and analyzed with FlowJo.

### Cytokines, metabolites and NAD and NADH measurement

Culture supernatant from BMDMs was collected as indicated in the experiments and the cytokine concentrations were measured by commercial ELISA kits according to the manufacturer’s instructions (BioVision). Lactate levels in media were quantified by using a lactate lit (K607 Lactate Colorimetric/Fluorometric Assay Kit, BioVision). The intracellular concentrations of acetyl-coA, citrate and pyruvate in BMDMs were detected by using the PicoProbe Acetyl-coenzyme A assay kit (ab87546, Abcam), the citrate assay kit (ab83396, Abcam) and the Pyruvate assay kit (K609, BioVision), respectively. For NAD and NADH measurement, total NAD and NADH levels were measured using a NAD/NADH Quantitation Colorimetric Kit (K337, BioVision). The ratios of NAD^+^ (NAD–NADH) to NADH were calculated. All experiments were performed at least three times and the data were normalized by the cell numbers or protein contents.

### Histone protein extraction and immunoblotting

Whole-cell lysates were prepared by using RIPA Lysis Buffer (Thermo Fisher) with proteinase inhibitor cocktail (Thermo Fisher) and phosphatase-inhibitor cocktail (Thermo Fisher Scientific) as described previously^43^. For histone extraction, cells were resuspended in lysis buffer (PBS containing 0.5% Triton X-100 (vol/vol), 2 mM phenylmethyl sulfonyl fluoride, 10 mm sodium butyrate and protease-inhibitor cocktail), incubated on ice for 10 min, and centrifuged at 6,500*g* for 10 min to enrich nuclei. The nuclei pellet was resuspended with 0.2 N HCl overnight at 4 °C for extraction of histones. Protein concentration was determinate by using Bradford reagent (Bio-Rad). For immunoblotting, primary antibodies used were anti-ACLY (CST, 4332; 1:2,000 dilution), anti-CPT1A (ab128568; 1:1,000 dilution) anti-LDHA (CST no. 2012; 1:3,000 dilution), anti-GLS (Thermo Fisher, 701965; 1:1,000 dilution), anti-MDH1 (ab180152; 1:3,000 dilution), anti-AMPK alpha 1 (ab32047; 1:1,000 dilution), anti-phospho-AMPKα (Thr172; CST, 4188; 1:1,000 dilution), anti-glutaminase (Thermo Fisher 701965; 1:1,000 dilution), anti-β-actin (Sigma A5441; 1:20,000 dilution), anti-acetyl-histone H3 (Millipore, 06-599; 1:5,000 dilution), anti-acetyl-histone H4 (Millipore, 06-866; 1:3,000 dilution) and anti-histone H3 (acetyl K27; ab4729; 1:3,000 dilution), anti-histone H3 (C terminus; BioLegend, 819411; 1:100 dilution) and anti-histone H4 (D2X4V; CST, 13919; 1:2,000 dilution).

### Flow cytometry-based metabolic analyses and Seahorse assays

Cells were washed and incubated with DMEM containing 100 nM MG (Thermo Fisher Scientific) and 20 nM TMRM (Thermo Fisher Scientific) for 30 min at 37 °C for measuring mitochondrial membrane potential and mass, respectively. After staining, the cells were washed and resuspended in FACS buffer (PBS containing 2% FBS) for surface marker staining as described above. FACS analyses were performed using an Attune NxT Flow Cytometer (Thermo Fisher Scientific). Data were analyzed using FlowJo. For measuring uptake of fatty acids and lipid content, cells were stained with 0.5 μg ml^−1^ BODIPY-FL C12 (Thermo Fisher, C3927MP) or 200 ng ml^−1^ BODIPY-493/503 (Thermo Fisher, D3922) in DMEM for 15 min at 37 °C. For extracellular flux assay, 3 × 10^5^ BMDMs were plated in a Seahorse Bioscience culture plate for overnight. Cells were then activated for 18 h with indicated treatments. OCR and ECAR were measured by an XF24 Seahorse Extracellular Flux Analyzer following the manufacturer’s instruction. In seahorse assays, BMDMs were treated with oligomycin (4 μM), FCCP (1.6 μM), rotenone (0.5 μM), antimycin A (0.5 μM) or etomoxir (18 μM). Each condition was performed with 3–4 replicates, and the readings of OCR and ECAR of each well were normalized to protein amount.

### Tumor engraftment and FGK45 treatment in vivo

For tumor engraftment, 1 × 10^5^ YUMM1.7 tumor cells were injected subcutaneously in 50 μl PBS. Tumors were measured via a caliper every 3 d after tumor engraftment with or without the indicated treatments and tumor volume was calculated by volume = (length × width^2^)/2. For FGK45 treatment, tumor-bearing mice were treated with monoclonal agonistic CD40 antibody (100 μg per injection, clone FGK45, Bio X cell) by intraperitoneal injection every 3 d from day 7 after tumor implantation.

### ^13^C-labeled palmitic acid and glutamine incorporation on histone H3

BMDMs cultured with ^13^C-labeling palmitic acid or glutamine were treated with or without FGK45 before extracting histones as described before. Dried histone samples were redissolved in 50 μl of HEPES buffer (200 mM, pH 8.5). Propionylation was carried out by adding 8 μl of freshly prepared propionylation reagent (150 μl of 2-propanol + 50 μl propionic anhydride) to 12 μl of histone solution and incubating at 37 °C with gentle shaking. After 3 min, 5 μl of fresh propionylation reagent were added and the reaction was continued for a total of 20 min. Samples were frozen in liquid nitrogen, dried by evaporation and then resuspended in 100 μl of ammonium bicarbonate (100 mM, pH 7.8). The final pH was then adjusted to 8.0 if needed by adding 0.5-μl aliquots of unbuffered Tris base (1.0 M). Samples were sonicated in a bath sonicator for 20 s then incubated at room temperature for 20 min with gentle agitation. To quench rests of propionylation reagent, 3 μl of 50% (vol/vol) hydroxylamine was added, followed by 20 min of further incubation at room temperature. Digestion was carried out by adding 0.5 μg modified trypsin (Promega) and incubating at 37 °C for 3 h under agitation. Samples were acidified by adding 10 μl of 1% TFA and 500 μl of 2% MeCN and clarified by centrifugation. Peptides were then desalted on a SepPak tC18 plate (Waters Corp) and eluted with 150 μl of 60% acetonitrile (MeCN). Dried eluates were resuspended in 30 μl of 2% MeCN, 0.01% TFA for HPLC–MS analysis. Samples were injected (5 μl) on a Q-Exactive Plus mass spectrometer interfaced via nanospray source to an Ultimate 3000 RSLCnano HPLC system (Thermo Scientific). After loading onto a trapping microcolumn (Acclaim PepMap100 C18, 20 mm × 100 μm ID, 5 μm, Thermo Scientific), peptides were separated on a reversed-phase custom-packed 40 cm C_18_ column (75 μm ID, 100 Å, Reprosil Pur 1.9-μm particles, Dr. Maisch, Germany) with a 7–25% MeCN gradient in 0.1% formic acid in 27 min, followed by a ramp up to 45% in 5 min at a flow rate of 0.25 μl min^−1^. Full mass spectrometry survey scans were performed at a resolution of 70,000. In data-dependent acquisition controlled by Xcalibur 3.0.63 software (Thermo Scientific), the ten most intense multiply charged precursor ions detected in the full spectrometry survey scan were selected for higher energy collision-induced dissociation (HCD, normalized collision energy NCE = 27%) and analyzed in the orbitrap at a resolution of 17,500. The window for precursor isolation was of 1.5 *m/z* units around the precursor and selected fragments were excluded for 60 s from further analysis. After data analysis and identification of precursors of interest, samples were reanalyzed with a modified method including a targeted SIM scan from a *m/z* of 525–540 at a resolution of 70,000 and with a target accumulation of 1E6 ions in a maximum of 100 ms.

### Tracing of metabolic fate of ^13^C-labeled palmitic acid and glutamine

BMDMs cultured with ^13^C-labeled palmitic acid or glutamine were treated with or without FGK45 as described before. Metabolites were extracted on dry ice/ice with 800 µl cold 62.5% methanol in water, containing norvaline and glutarate as internal standards. Cells were scraped and 500 µl ice-cold chloroform was added. The samples were vortexed at 4 °C to extract metabolites, centrifuged for 10 min and the phases were separated. For measurements of polar metabolites, the methanol water phase was dried at 4 °C by vacuum centrifugation. For GC–MS analysis, samples were derivatized. Methoxyamine in pyridine (Sigma) was added and samples were incubated at 37 °C for 90 min, *N*-(tert-butyldimethylsilyl)-N-methyl-trifluoroacetamide (TBDMS) was added for 60 min at 60 °C. Separation occurred with an Agilent 7890A GC system coupled to an Agilent 5975C Inert MS system. A DB35MS column was used and helium was selected as a carrier gas at a flow rate of 1 ml min^−1^. A volume of 1 µl of sample was injected in splitless mode with an inlet temperature of 270 °C. The GC oven was kept at 100 °C for 1 min, increased up to 105 °C with a gradient of 2.5 °C min^−1^, then ramped to 240 °C with a gradient of 3.5 °C min^−1^, and after that ramped up to 320 °C with a gradient of 2.5 °C min^−1^, which was followed by 4 min at 320 °C. Mass spectrometry was performed at 70 eV and a mass range of 100–650 atomic mass units was measured. Isotopolog distributions of the metabolites were quantified, normalized to protein and corrected for natural abundance with a MATLAB script.

### Data analysis of histone labeling

Tandem mass spectrometry data from data-dependent acquisition runs were analyzed with MASCOT 2.8 (Matrix Science). Mascot was set up to search the UNIPROT database (SWISSPROT + TrEMBL; https://www.expasy.org/) restricted to *Mus musculus* taxonomy. The database release used was June 2020, containing 17,063 sequences after taxonomy filter. Trypsin (cleavage at K and R, not before P) was used as the enzyme definition with a maximum of three missed cleavages. Mascot searches were done with a fragment ion mass tolerance of 0.02 Da and a parent ion tolerance of 10 ppm. The iodoacetamide derivative of cysteine was specified in Mascot as a fixed modification. Propionylation, trimethylation and acetylation were specified as variable modifications on lysine, with delta masses of +56.026215, +42.04695 and +42.010565 atomic mass units, respectively. Propionylation was also allowed on peptide N termini, to account for spurious reactions. After identifications of precursors of interest and their retention times, integrated peak areas were extracted using the software Freestyle (Thermo Fisher Scientific) with a mass tolerance of 8 ppm. The traces of the monoisotopic (C_13_(0)) and A + 2 peaks (C_13_(2)) were integrated and used as a simplified measure of heavy isotope incorporation. The natural relative intensity of the C_13_(2) isotopomer without enrichment was calculated with the QualBrowser software (Thermo Fisher Scientific) and used as a reference.

### Statistics

No statistical methods were used to predetermine sample sizes but our sample sizes are similar to those reported in previous publications^12^. Mice were randomized in the experiments and data distribution was assumed to be normal but this was not formly tested. Data collection and analysis were not performed blind to the conditions of the experiments and we did not exclude any participants from the analysis. All results are presented as the mean ± s.d. Statistical significance was performed using GraphPad Prism version 7 and unpaired Student’s *t*-tests. *P* values and nonsignificant values are labeled in the figures. *P* < 0.05 was considered statistically significant.

### Reporting summary

Further information on research design is available in the Nature Portfolio Reporting Summary linked to this article.

## Online content

Any methods, additional references, Nature Portfolio reporting summaries, source data, extended data, supplementary information, acknowledgements, peer review information; details of author contributions and competing interests; and statements of data and code availability are available at 10.1038/s41590-023-01430-3.

### Supplementary information

Supplementary Information Supplementary Tables 1–3.

Reporting Summary

### Source data

Source Data Fig. 1 Source data for graphs in Fig. 1.

Source Data Fig. 2 Source data for graphs in Fig. 2.

Source Data Fig. 3 Source data for graphs in Fig. 3.

Source Data Fig. 4 Source data for graphs in Fig. 4.

Source Data Fig. 5 Source data for graphs in Fig. 5.

Source Data Fig. 6 Source data for graphs in Fig. 6.

Source Data Uncropped immunoblots.

Source Data Extended Data Fig. 1 Source data for graphs in Extended Data Fig. 1.

Source Data Extended Data Fig. 2 Source data for graphs in Extended Data Fig. 2.

Source Data Extended Data Fig. 3 Source data for graphs in Extended Data Fig. 3.

Source Data Extended Data Fig. 4 Source data for graphs in Extended Data Fig. 4.

Source Data Extended Data Fig. 5 Source data for graphs in Extended Data Fig. 5.

Source Data Extended Data Fig. 6 Source data for graphs in Extended Data Fig. 6.

Source Data Extended Data Fig. 7 Source data for graphs in Extended Data Fig. 7.

## Data Availability

The data generated in this study are available in the main article and Supplementary Information or are available from the corresponding authors upon reasonable request. Source data are provided with this paper.
