# Author Correction: CD40 signal rewires fatty acid and glutamine metabolism for stimulating macrophage anti-tumorigenic functions

**DOI:** 10.1038/s41590-023-01611-0

**Published:** 2023-08-10

**Authors:** Pu-Ste Liu, Yi-Ting Chen, Xiaoyun Li, Pei-Chun Hsueh, Sheue-Fen Tzeng, Hsi Chen, Pei-Zhu Shi, Xin Xie, Sweta Parik, Mélanie Planque, Sarah-Maria Fendt, Ping-Chih Ho

**Affiliations:** 1 Institute of Cellular and System Medicine, National Health Research Institute, Miaoli, Taiwan; 2 Department of Fundamental Oncology, Faculty of Biology and Medicine, University of Lausanne, Lausanne, Switzerland; 3 Ludwig Lausanne Branch, Lausanne, Switzerland; 4 School of Life Science, Shaoxing University, Shaoxing, People’s Republic of China; 5 Laboratory of Cellular Metabolism and Metabolic Regulation, Center for Cancer Biology, VIB, Leuven, Belgium; 6 Laboratory of Cellular Metabolism and Metabolic Regulation, Department of Oncology, KU Leuven and Leuven Cancer Institute (LKI), Leuven, Belgium

**Keywords:** Biochemistry, Tumour immunology, Monocytes and macrophages

Correction to: *Nature Immunology* 10.1038/s41590-023-01430-3. Published online 23 February 2023.

In the initially published version of this article, the *x*-axis labels of Extended Data Fig. 1d, now reading “FGK45” and “Ctrl” left to right, were switched. The error has been corrected in the HTML and PDF versions of the article.

