# Supplementary material for: CD40 signal rewires fatty acid and glutamine metabolism for stimulating macrophage anti-tumorigenic functions
Source: Nat Immunol. 2023 Feb 23;24(3):452–62. doi: 10.1038/s41590-023-01430-3 (PMC9977680; doi:10.1038/s41590-023-01430-3)
Supplement: Supplementary file 1 — Supplementary Tables 1–3. [file 41590_2023_1430_MOESM1_ESM.pdf]

nature immunology

Article https://doi.org/10.1038/s41590-023-01430-3

CD40 signal rewires fatty acid and glutamine metabolism for stimulating macrophage anti-tumorigenic functions

In the format provided by the authors and unedited

nature immunology

Article https://doi.org/10.1038/s41590-023-01430-3

CD40 signal rewires fatty acid and glutamine metabolism for stimulating macrophage anti-tumorigenic functions

In the format provided by the authors and unedited

Supplementary Table 1. Sequence for sgRNAs for CRISPR-based gene targeting

Gene name	sgRNA targeting sequence
Cpt1a sgRNA-1	GCGGAGATCGATGCCATCAG
Cpt1a sgRNA-2	GCCTGTCAGGGCTGCACTCC
LDHA sgRNA-1	CTCAGGCGGCTACACGTACA
LDHA sgRNA-2	CGGGGGCCCGTCAGCAAGAG
ACLY sgRNA-1	GCTTCGTCCCCCACAGTCAGGCGG
ACLY sgRNA-2	GGACCAGTTAATCAAACGTCGAGG
GLS sgRNA-1	GAAAGTGCTAAAAAGCAGTC
GLS sgRNA-2	GGCGGTATGATGCGGCTGCG

Supplementary Table 2. Primer sequence for qPCR analysis

Gene name	Primer sequence for qPCR
Il1b-F	GCAACTGTTCCTGAACTCAACT
Il1b-R	ATCTTTTGGGGTCCGTCAACT
Tnfa-F	ACGGCATGGATCTCAAAGAC
Tnfa-R	AGATAGCAAATCGGCTGACG
Il6-F	TAGTCCTTCCTACCCCAATTTCC
Il6-R	TTGGTCCTTAGCCACTCCTTC
IL12-F	AATGTCTGCGTGGAAGCTCA
IL12-R	ATGCCCACTTGCTGCATGA
actin-F	TCCATCATGAAGTGTGACGT
actin-R	TACTCCTGCTTGCTGATCCAC
Arg1-F	CTCCAAGCCAAAGTCCTTAGAG
Arg1-R	AGGAGCTGTCATTAGGGACATC
Mrc1-F	CTCTGTTCAGCTATTGGACGC
Mrc1-R	CGGAATTTCTGGGATTCAGCTTC
Ym1-F	AGAAGGGAGTTTCAAACCTGGT
Ym1-R	GTCTTGCTCATGTGTGTAAGTGA
Retnla-F	CTGGGTTCTCCACCTCTTCA
Retnla-R	TGCTGGGATGACTGCTACTG

Supplementary Table 3. Primers sequence for ChIP analysis

Gene name	Primer sequence for qPCR
Il1b-F	CACAGAAGCACCATCCAGT
Il1b-R	AGATGCACACCCAGAAGTG
Il6-F	TCCCATCAAGACATGCTCAAGTGC
Il6-R	AGCAGAATGAGCTACAGACATCCC
IL12-F	TACCTTACATTTGAGTGATGGACTTCT
IL12-R	TCAACTTTTTCTTTCTGTGTGACATAATTTA
