# Supplementary material for: CD40 signal rewires fatty acid and glutamine metabolism for stimulating macrophage anti-tumorigenic functions
Source: Nat Immunol. 2023 Feb 23;24(3):452–62. doi: 10.1038/s41590-023-01430-3 (PMC9977680; doi:10.1038/s41590-023-01430-3)
Supplement: Supplementary file 2 — Reporting Summary [file 41590_2023_1430_MOESM2_ESM.pdf]

nature research

Corresponding author(s): NI-A34202

Last updated by author(s): Dec 19, 2022

Reporting Summary

Nature Research wishes to improve the reproducibility of the work that we publish. This form provides structure for consistency and transparency in reporting. For further information on Nature Research policies, see our Editorial Policies and the Editorial Policy Checklist.

Statistics

For all statistical analyses, confirm that the following items are present in the figure legend, table legend, main text, or Methods section.

n/a	Confirmed	
☐	☒	The exact sample size (*n*) for each experimental group/condition, given as a discrete number and unit of measurement
☐	☒	A statement on whether measurements were taken from distinct samples or whether the same sample was measured repeatedly
☐	☒	The statistical test(s) used AND whether they are one- or two-sided<br>*Only common tests should be described solely by name; describe more complex techniques in the Methods section.*
☒	☐	A description of all covariates tested
☐	☒	A description of any assumptions or corrections, such as tests of normality and adjustment for multiple comparisons
☐	☒	A full description of the statistical parameters including central tendency (e.g. means) or other basic estimates (e.g. regression coefficient) AND variation (e.g. standard deviation) or associated estimates of uncertainty (e.g. confidence intervals)
☐	☒	For null hypothesis testing, the test statistic (e.g. *F*, *t*, *r*) with confidence intervals, effect sizes, degrees of freedom and *P* value noted<br>*Give P values as exact values whenever suitable.*
☒	☐	For Bayesian analysis, information on the choice of priors and Markov chain Monte Carlo settings
☒	☐	For hierarchical and complex designs, identification of the appropriate level for tests and full reporting of outcomes
☒	☐	Estimates of effect sizes (e.g. Cohen's *d*, Pearson's *r*), indicating how they were calculated

Our web collection on statistics for biologists contains articles on many of the points above.

Software and code

Policy information about availability of computer code

Data collection	Attune NxT Flow Cytometer (ThermoFisher Scientific)<br>BD Influx (BD Biosciences)<br>Ultimate 3000 RSLCnano HPLC system (Thermo Scientific)<br>Agilent 7890A GC system<br>Agilent 5975C Inert MS system
Data analysis	Attune™ NxT Software<br>Flowjo v10.4<br>GraphPad PRISM version 7<br>Xcalibur 3.0.63 software (Thermo Scientific)<br>MASCOT 2.8 (Matrix Science, London, UK)<br>QualBrowser software (Thermo Fisher Scientific)

For manuscripts utilizing custom algorithms or software that are central to the research but not yet described in published literature, software must be made available to editors and reviewers. We strongly encourage code deposition in a community repository (e.g. GitHub). See the Nature Research guidelines for submitting code & software for further information.

Data

Policy information about availability of data

All manuscripts must include a data availability statement. This statement should provide the following information, where applicable:

- Accession codes, unique identifiers, or web links for publicly available datasets
- A list of figures that have associated raw data
- A description of any restrictions on data availability

All data are present in the article and supplementary information files are available from the corresponding authors upon reasonable request.

Field-specific reporting

Please select the one below that is the best fit for your research. If you are not sure, read the appropriate sections before making your selection.

☒ Life sciences ☐ Behavioural & social sciences ☐ Ecological, evolutionary & environmental sciences

For a reference copy of the document with all sections, see nature.com/documents/nr-reporting-summary-flat.pdf

Life sciences study design

All studies must disclose on these points even when the disclosure is negative.

Sample size	The exact sample size of all in vivo experiments were reported in the figure legends section. All in vitro experiment had at least 3 biological replicates in each group. No statistical methods were used to pre-determine sample size.
Data exclusions	No data exclusions
Replication	Biological replicates were used in all experiments and descripted in each figure legends.
Randomization	For all mice experiments, groups were randomized and selected based on mouse genotype, gender and weight.
Blinding	Investigators were not blinded. Blinding was not possible as mouse genotype and cells were screened before groups setup

Reporting for specific materials, systems and methods

We require information from authors about some types of materials, experimental systems and methods used in many studies. Here, indicate whether each material, system or method listed is relevant to your study. If you are not sure if a list item applies to your research, read the appropriate section before selecting a response.

Materials & experimental systems

n/a	Involved in the study	
☐	☒	Antibodies
☐	☒	Eukaryotic cell lines
☒	☐	Palaeontology and archaeology
☐	☒	Animals and other organisms
☒	☐	Human research participants
☒	☐	Clinical data
☒	☐	Dual use research of concern

Methods

n/a	Involved in the study	
☒	☐	ChIP-seq
☐	☒	Flow cytometry
☒	☐	MRI-based neuroimaging

Antibodies

Antibodies used

For in vitro FGK45 stimulation, FGK45(Bio X Cell Catalog #BP0016-2, anti-mouse CD40 monoclonal antibody) was crosslinked with goat anti-rat immunoglobulin G (10ng/ml, biolegend, cat 405401)
For immunoblotting, primary antibodies used were anti-ACLY (CST, cat# 4332), anti-CPT1A antibody(ab128568), anti-LDHA antibody(CST#2012), anti-GLS antibody (Thermo Fisher #701965), anti-MDH1antibody (ab180152), anti-AMPK alpha 1(ab32047), anti-Phospho-AMPKα (Thr172; CST #4188), anti-glutaminase antibody (Thermo Fisher 701965), anti- β actin (Sigma A5441), anti-acetyl-Histone H3 (Millipore 06-599), anti-acetyl-Histone H4 (Millipore 06-866) and anti-Histone H3 (acetyl K27) antibody (ab4729), anti-Histone H3 (C-terminus) antibody(Biolengend 819411) and anti-Histone H4 (D2X4V; CST, cat#13919).
For immunoblotting, antibodies used were anti-acetyl-Histone H3 (Millipore 06-599), anti-acetyl-Histone H4 (Millipore 06-866) and anti-Histone H3 (acetyl K27) antibody (Abcam 4729), or IgG (Santa Cruz, Dallas, TX, SC-2027) antibodies.
FACS antibodies were used from BioLegend: Brilliant Violet 650™ anti-mouse CD45 Antibody (30-F11; Cat. no. 103151), Pacific blue

	anti-mouse/human CD11b Antibody (M1/70; Cat. no. 101224), PE/Cy7 anti-mouse Ly-6G/Ly-6C (Gr-1) Antibody (RB6-8C5; Cat. no. 108416), APC-C7 anti-mouse F4/80 Antibody (BM8; Cat. no. 123116)
Validation	All commercial available antibodies were validated by the manufacturer in their product sheet and antibody information were available by searching their catalog number on the manufacturer website.

Eukaryotic cell lines

Policy information about cell lines

Cell line source(s)	The original cell line YUMM1.7 melanoma cell line was provided by Marcus Bosenberg.(ATCC)<br>L929 cells were purchased from ATCC.
Authentication	None of the cell lines were authenticated in these studies, but all cells were used at low passage numbers.
Mycoplasma contamination	All the cell lines are mycoplasma-free. They have been tested for mycoplasma contamination regularly.
Commonly misidentified lines (See ICLAC register)	No commonly misidentified cell lines were used.

Animals and other organisms

Policy information about studies involving animals; ARRIVE guidelines recommended for reporting animal research

Laboratory animals	In all animal experiments, animals (6-8 weeks old) were sex-matched within each experiment and both sexes were used. C57BL/6/J mice, LDHAflox mice, LysM-Cre mice and Rosa26-Cas9 knock in mice were purchased from The Jackson Laboratory. Macrophages specific knockout Cpt1a, ACLY or Gls mice were generated by using bone marrow transplantation. Mice were housing in conventional animal facility of the University of Lausanne or National Health Research Institutes laboratory animal center National Health Research Institutes laboratory animal center and keep in individually ventilated cages within animal rooms maintained on standard 12-h light/dark cycles and an ambient temperature of 19-23 °C.
Wild animals	No wild animals were involved
Field-collected samples	No samples were collected from the field
Ethics oversight	All animal experiments were approved and performed in accordance with guidelines and regulations of the Institutional Animal Care and Use Committees of National Health Research Institutes laboratory animal center or Swiss federal regulations and approved by the veterinary authority of Canton Vaud.

Note that full information on the approval of the study protocol must also be provided in the manuscript.

Flow Cytometry

Plots

Confirm that:

☐ The axis labels state the marker and fluorochrome used (e.g. CD4-FITC).

☒ The axis scales are clearly visible. Include numbers along axes only for bottom left plot of group (a 'group' is an analysis of identical markers).

☒ All plots are contour plots with outliers or pseudocolor plots.

☒ A numerical value for number of cells or percentage (with statistics) is provided.

Methodology

Sample preparation	Cells were harvested from tumors using mechanical homogenization and digestion using 2% FBS, DNase I (1 µg/ ml, Sigma-Aldrich) and collagenase IV (0.5 mg/ml, Sigma-Aldrich) at 37 °C for 45 min. Cells were filtered through a 70-µm cell strainer and leukocyte enrichment was separated by density gradient centrifugation (800g, 30 min) at 25 °C with 40% and 80% Percoll (GE Healthcare). For BMDM, cells were collected, washed in FACS buffer before staining.
Instrument	Cell were analyzed by Attune NxT Flow Cytometer (ThermoFisher Scientific). Sorting cells were using BD Influx (BD Biosciences) the 140µm nozzle and 7.5 psi pressure, to a purity of ~95–99%.
Software	Flowjo v10.4 was used for data analysis.
Cell population abundance	All analysis have been performed on a population that included between 10000 to 20000 macrophages within tumor and spleen samples, depending on initial material abundance.

Gating strategy

We first used FSC/SSC and LIVE/DEAD Fixable Violet Cell Stain Kit to gate live and single cells. Then, these cells were gated based on the analysis performed : TAM and splenic macrophages were identified based on the expression markers : CD45+ Cd11b+Gr1- F4/80+ from tumors. BMDM cells were selected based on F4/80 and Cd11b positive population.

☒ Tick this box to confirm that a figure exemplifying the gating strategy is provided in the Supplementary Information.
