# Supplementary material for: CD40 signal rewires fatty acid and glutamine metabolism for stimulating macrophage anti-tumorigenic functions
Source: Nat Immunol. 2023 Feb 23;24(3):452–62. doi: 10.1038/s41590-023-01430-3 (PMC9977680; doi:10.1038/s41590-023-01430-3)

Source Fig. 3c

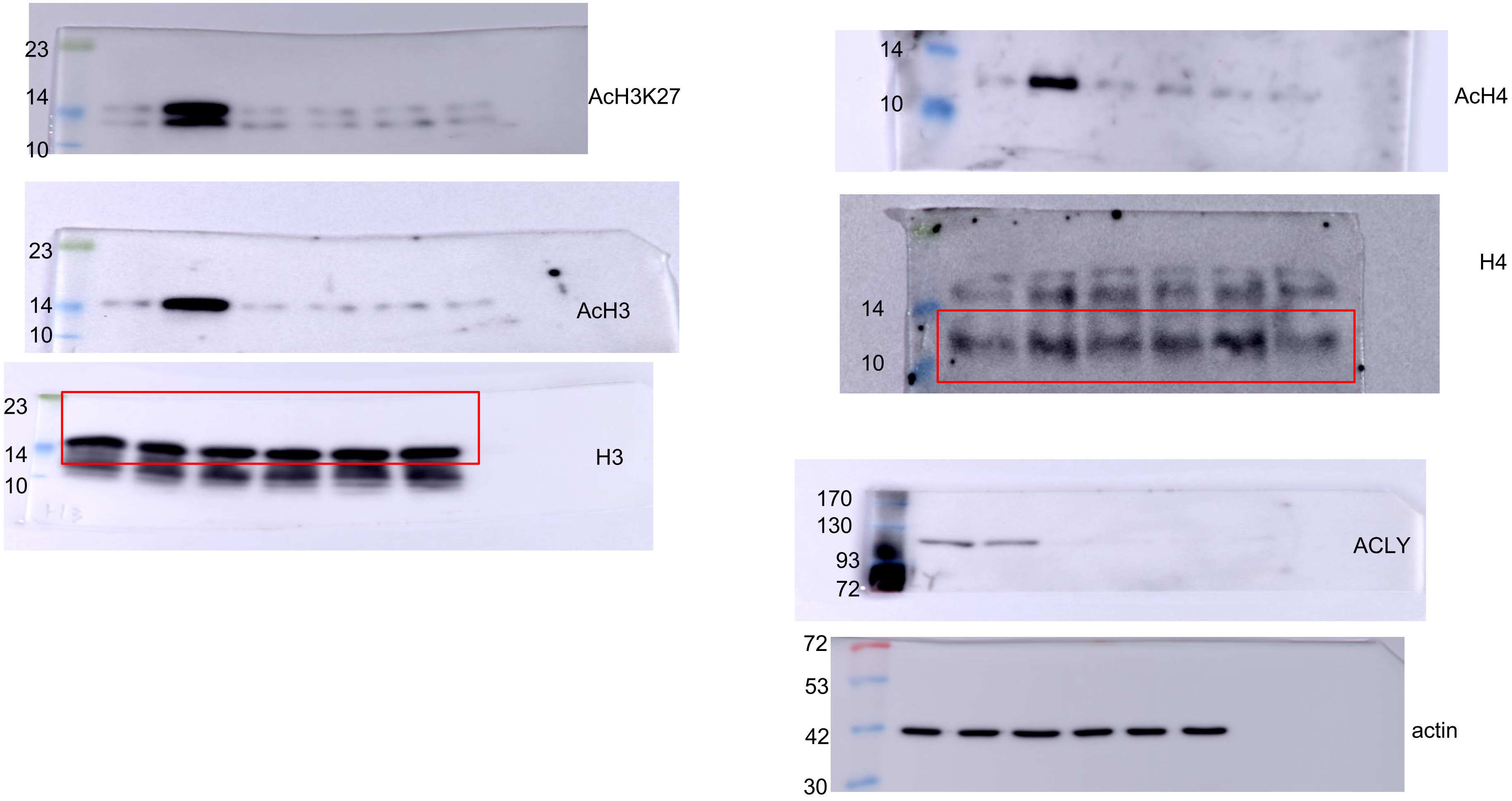

Source Fig. 4h

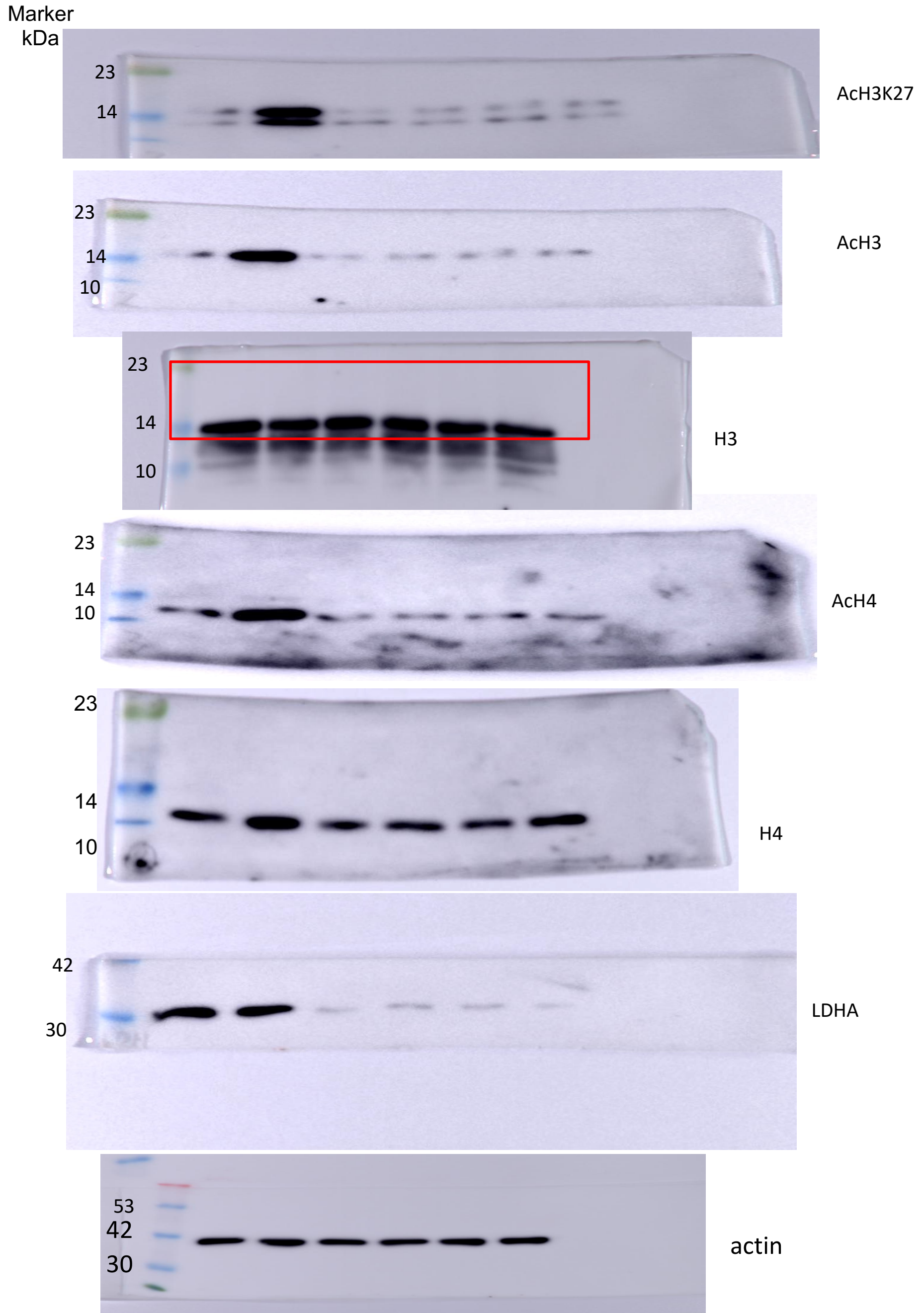

Source Fig. 4l

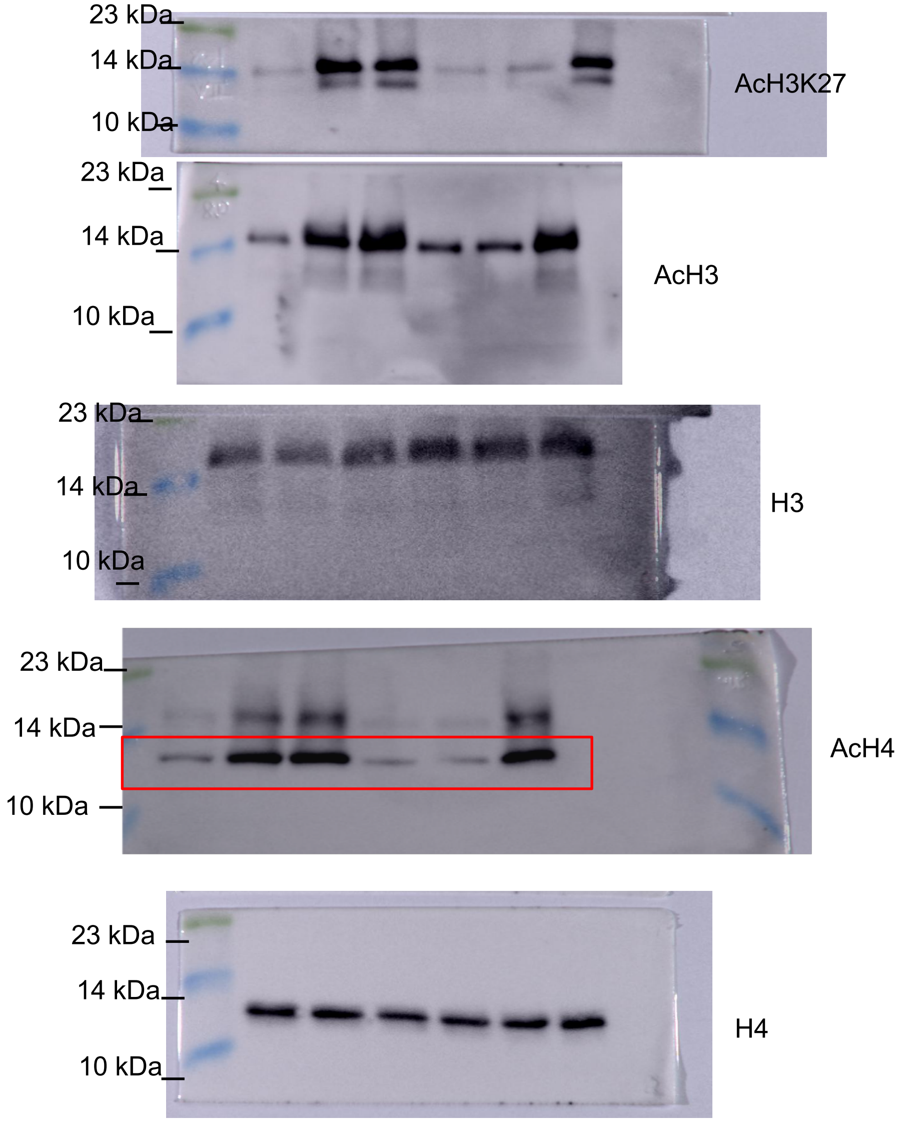

Source Fig. 6b

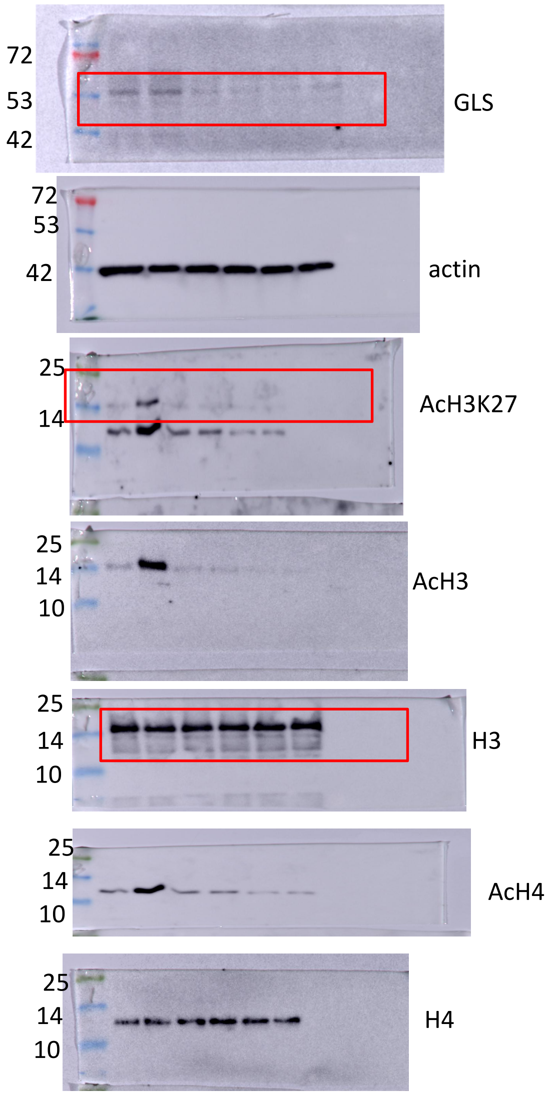

Source Fig. 6o

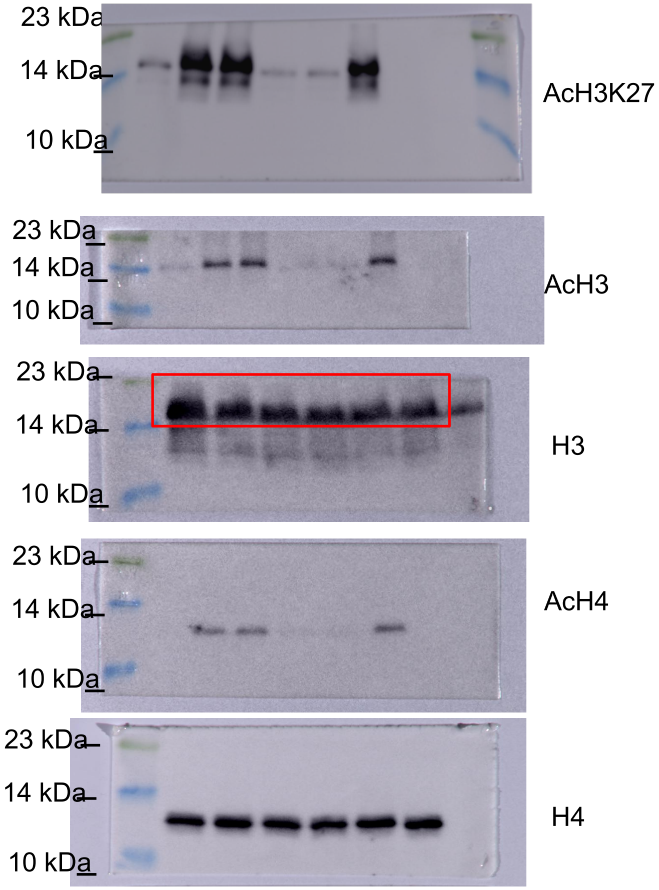

Extended Data Figure 3d

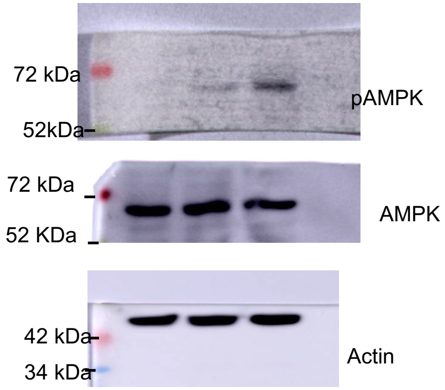

Extended Data Figure 3b

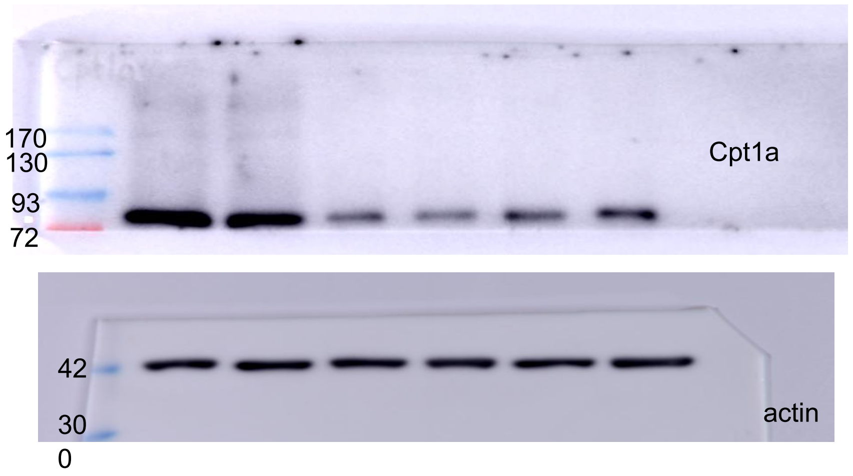

Extended Data Figure 5h

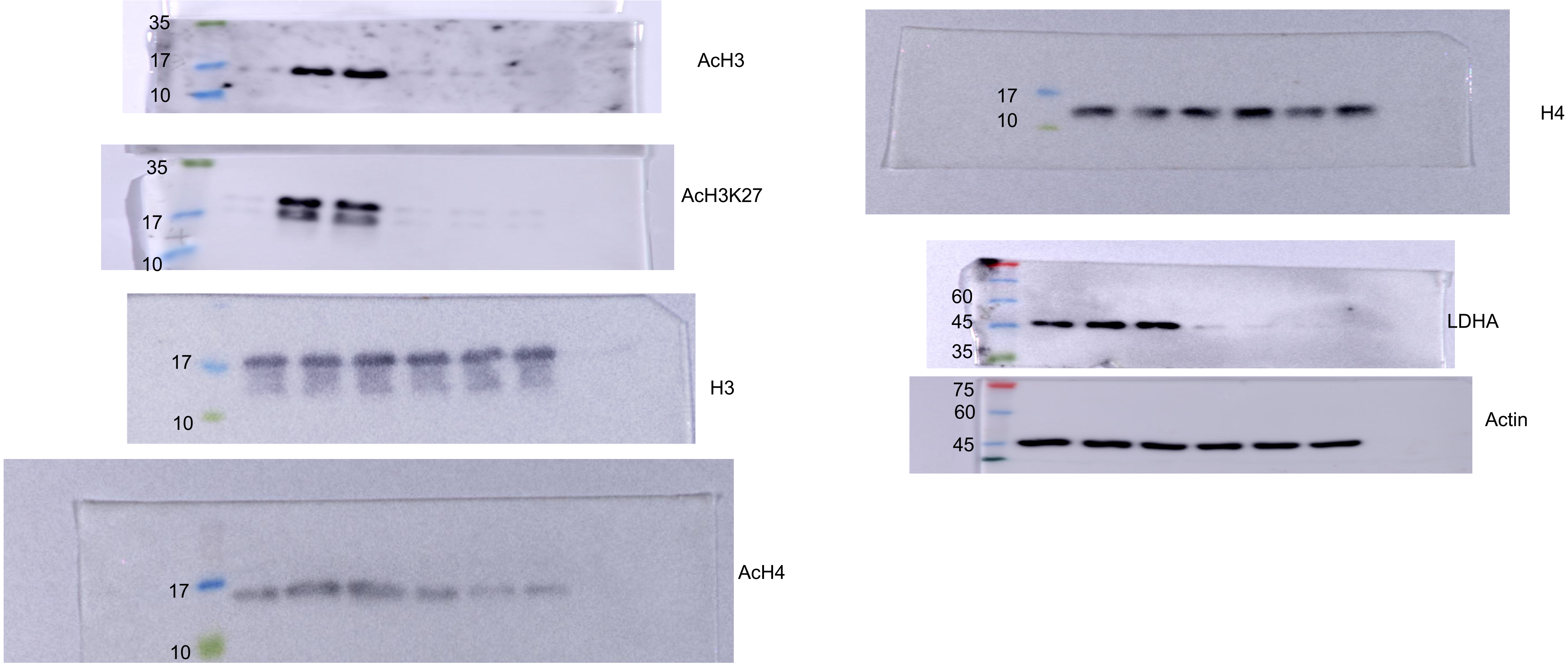

Extended Data Figure 6g

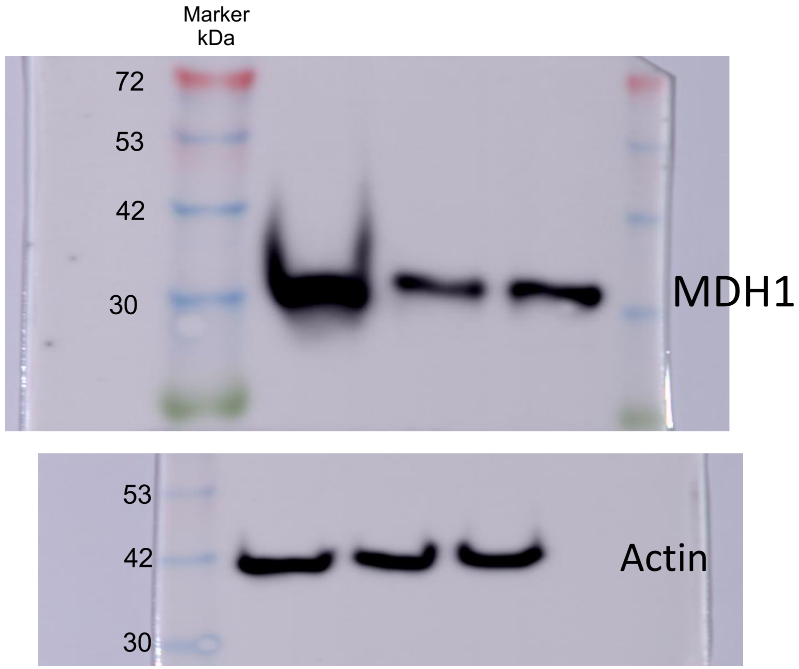

Supplement: Supplementary file 9 — Uncropped immunoblots. [file 41590_2023_1430_MOESM9_ESM.pdf]
